# A Revision of Malagasy Species of *Anochetus* Mayr and *Odontomachus* Latreille (Hymenoptera: Formicidae)

**DOI:** 10.1371/journal.pone.0001787

**Published:** 2008-05-28

**Authors:** Brian L. Fisher, M. Alex Smith

**Affiliations:** 1 Department of Entomology, California Academy of Sciences, San Francisco, California, United States of America; 2 Biodiversity Institute of Ontario, University of Guelph, Guelph, Ontario, Canada; Missouri Botanical Garden, United States of America

## Abstract

Species inventories are essential for documenting global diversity and generating necessary material for taxonomic study and conservation planning. However, for inventories to be immediately relevant, the taxonomic process must reduce the time to describe and identify specimens. To address these concerns for the inventory of arthropods across the Malagasy region, we present here a collaborative approach to taxonomy where collectors, morphologists and DNA barcoders using cytochrome c oxidase 1 (CO1) participate collectively in a team-driven taxonomic process. We evaluate the role of DNA barcoding as a tool to accelerate species identification and description.

This revision is primarily based on arthropod surveys throughout the Malagasy region from 1992 to 2006. The revision is based on morphological and CO1 DNA barcode analysis of 500 individuals. In the region, five species of *Anochetus* (*A. boltoni* **sp. nov.**, *A. goodmani* **sp. nov.**, *A. grandidieri*, and *A. madagascarensis* from Madagascar, and *A. pattersoni* **sp. nov.** from Seychelles) and three species of *Odontomachus* (*O. coquereli, O. troglodytes* and *O. simillimus*) are recognized. DNA barcoding (using cytochrome *c* oxidase 1 (CO1)) facilitated caste association and type designation, and highlighted population structure associated with reproductive strategy, biogeographic and evolutionary patterns for future exploration.

This study provides an example of collaborative taxonomy, where morphology is combined with DNA barcoding. We demonstrate that CO1 DNA barcoding is a practical tool that allows formalized alpha-taxonomy at a speed, detail, precision, and scale unattainable by employing morphology alone.

## Introduction

*Anochetus* and *Odontomachus* were treated globally by Brown [1], [2]. This paper revises the genera for the Island of Madagascar and also includes new records from the Seychelles and Comoro Islands. The revision is based on morphological and CO1 sequence analysis of 500 individuals. We evaluate the role of DNA barcoding as a tool to accelerate species identification and description.

**Figure 1:**
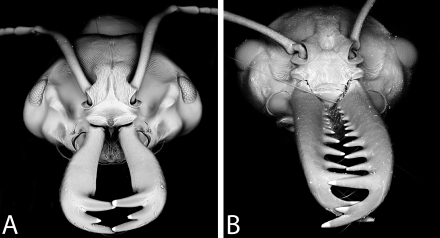
oblique dorsal view of head. A, *Anochetus madagascarensis*. B, *Odontomachus coquereli*.

*Anochetus* and *Odontomachus* are closely related genera [1], [3], [4] characterized by long and straight mandibles inserted just on either side of the cephalic midline and with two or three large teeth near tip arranged in a vertical series (Figure 1a,b). The single tooth or spine at the apex of the petiole separates *Odontomachus* from the closely related genus *Anochetus* (which has two teeth or rounded margin). *Odontomachus* and *Anochetus* can also be easily distinguished by the characters on the back of the head. With head viewed from back near neck of pronotum, *Odontomachus* has dark, inverted V-shaped apophyseal lines that converge to form a distinct, sometimes shallow groove or ridge on upper back of head. In *Anochetus*, the V-shaped apophyseal lines are absent. In the same region of the back of head, however, nuchal carinae in *Anochetus* form an uninterrupted, inverted U-shaped ridge. In the field, small members of *Anochetus* might also be mistaken for *Strumigenys*, from which they may be distinguished by their one-segmented waist (vs. two segments in *Strumigenys*).

The utility of a standardized single gene for species recognition (but not phylogenetics) has been tested in an increasing swath of life. Here we tested how well a cytochrome *c* oxidase 1 (CO1) DNA barcode resolved species within Malagasy *Anochetus* and *Odontomachus*. In Madagascar, these ponerine genera are known to include species with independent colony formation by ergatoid (wingless) queens – and therefore are expected be a challenge for DNA barcoding using a single mitochondrial marker – but also include cases where prior taxonomy has not linked males with females and workers, nor has resolved obvious worker dimorphism as either caste variation or provisional species.

Species level taxonomy in these genera can be quite difficult. Brown [2] noted that males provide a useful source for species level delimitation. Males, however, are rarely associated with the worker castes. Brown [2:553] states: “Unfortunately, males found associated in the nest with the female castes are known only for a minority of the species. Additional kinds of males are known from collections at light or by Malaise trap, but it has not yet been possible to link any of these securely to worker-based species.”

In this study, we used CO1 barcode sequences to associate worker, queen and male castes. We conclude that DNA barcoding will enable species delimitation, linking a greater range of the morphological diversity in ants (castes and sex), and further will provide a set of molecular characters that improve species delimitation and identification while making these hypotheses transparent and reproducible.

## Methods

This revision is primarily based on arthropod surveys in Madagascar that included over 6,000 leaf litter samples, 4,000 pitfall traps, and 8,000 additional hand collecting events throughout Madagascar from 1992 through 2006 [5]. Also included are specimens from museums in Genoa, Geneva, Paris, London, Berlin, Tervuren, and Basel and the extensive collection of Gary D. Alpert located at the MCZC. Overall, this revision included the study of approximately 1,700 specimens of *Anochetus* and *Odontomachus* from 1014 recorded collecting events from throughout Madagascar with additional samples from Comoros and Seychelles. Roy Snelling (LACM) provided the records of *O. simillimus* from his work on the ants of Seychelles. Samples were selected for CO1 sequencing throughout the geographic range of each species. In total, 501 specimens were sequenced. Specimens examined from Madagascar are listed by increasing latitude within provinces.

All species and type material examined in this study have been imaged and are available on AntWeb (www.antweb.org). Material was deposited at the California Academy of Sciences, San Francisco (CASC); British Museum of Natural History, London (BMNH); and Museum of Comparative Zoology, Harvard University, Cambridge, Massachusetts (MCZC). All sequences, oligonucleotides and electropherograms are deposited in BOLD (www.barcodinglife.org), and sequence data has been deposited on Genbank.

In accordance with section 8.6 of the ICZN's International Code of Zoological Nomenclature, we have deposited copies of this article at the following five publicly accessible libraries: Natural History Museum, London, UK; American Museum of Natural History, New York, USA; Museum National d'Histoire Naturelle, Paris, France; Russian Academy of Sciences, Moscow, Russia; Academia Sinica, Taipei, Taiwan. The three new species names established herein have been prospectively registered in ZooBank [6]–[8], the official online registration system for the ICZN. The ZooBank publication LSID (Life Science Identifier) for the new species described herein can be viewed through any standard web browser by appending the LSID to the prefix “http://zoobank.org/”.

New specific names in this work are attributive genitive nouns and thus invariant. Each specimen discussed below is uniquely identified with a specimen-level code (e.g. CASENT0003099) affixed to each pin. In addition, each specimen may include a collection code, which is a field number that uniquely identifies collecting events (e.g. BLF01652). Collection codes, when available, are associated with a collector and follow the collector's name.

Digital color images were created using a JVC KY-F75 digital camera and Syncroscopy Auto-Montage (v 5.0) software. All measurements were taken at 80× power with a Leica MZ APO microscope using an orthogonal pair of micrometers, recorded to the nearest 0.001 mm, and rounded to two decimal places for presentation. When more than one specimen was measured, minimum and maximum measurements and indices are presented. Measurements follow those used by Brown [1], [2]. Abdominal segments are noted by “A” and the segment number, such as A2 for the petiole and A3 for the first gastral segment.

### Abbreviations used:

HL: Head length: measured in full-face view; maximum longitudinal length from the anteriormost portion of the projecting mandible joint (the dorsal socket where the mandible turns) to the midpoint of a line across the posterior margin. (male: including ocelli)
HW: Head width: *Anochetus*: maximum width of head; *Odontomachus*: HW (across upper eye margin): maximum width of head measured across posterior margin of eyes; HW (across vertex): maximum width of head measured across temporal prominences. In *O*. *coquereli*, which lacks temporal prominences, the measurement is taken across the part of the vertex at which the sides are nearly parallel near or a little behind the midlength of the head. (male: including eyes)
ML: Mandible length: The straight-line length of the mandible at full closure, measured in the same plane for which the HL measurement is taken (full face view), from the mandibular apex to the anterior clypeal margin, or to the transverse line connecting the anterior most points in those taxa where the margin is concave medially.
EL: Eye length: maximum length of eye as measured normally in oblique view of the head to show full surface of eye.
SL: Scape length: maximum chord length excluding basal condyle and neck.
WL: Weber's length (Mesosoma length): in lateral view of the mesosoma, diagonal length from posteroventral corner of propodeum to the farthest point on anterior face of pronotum, excluding the neck.
PW: Pronotum width: in dorsal view, maximum width of pronotum.
FL: Femur length: Maximum length of hind femur.
CI: Cephalic index: HW/HL×100.
SI: Scape index: SL/HW×100.
MI: Mandible Index: ML/HL×100

Specimens of *Anochetus* and *Odontomachus* were examined from the following collections:

BMNH: Natural History Museum, London, U. K.
CASC: California Academy of Sciences, San Francisco, CA, USA
LACM: Los Angles County Museum, Los Angeles, CA, USA
MCZC: Museum of Comparative Zoology, Harvard University, Cambridge, MA, USA
MHNG: Muséum d'Histoire Naturelle, Geneva, Switzerland
MNHN: Muséum National d'Histoire Naturelle, Paris, France
MRAC: Musée Royal de l'Afrique Centrale, Tervuren, Belgium
MSNG: Museo Civico de Historia Natural “Giacomo Doria”, Genoa, Italy
NHMB: Naturhistorisches Museum, Basel, Switzerland
PSWC: P. S. Ward Collection, University of California at Davis, CA, USA

### CO1 methods

Specimens were preserved in 95% ethanol in Madagascar and upon return to California were loaded into ScrewTop TrakMates® boxes (Matrix Technologies) and shipped to the University of Guelph. There, DNA was extracted from tissues rich in mitochondria (e.g. legs), employing primers with high universality, and amplifying a PCR product approximately 600 bp in length. Total genomic DNA extracts were prepared from small pieces (≤1 mm) of tissue using the NucleoSpin® 96 Tissue kit (Macherey-Nagel Duren, Germany), following the manufacturer's protocols. Extracts were resuspended in 30 µl of dH_2_O, and a 650base-pair (bp) region near the 5′ terminus of the CO1 gene was amplified following standard protocol [9]. Briefly, full length sequences were amplified using primers (LF1-ATTCAACCAATCATAAAGATATTGG and LR1-TGATTTTTTGGACATCCAGAAGTTTA [10]). In cases where a 650 bp product was not successfully generated, internal primer pairs (LF1–ANTMR1-(see Table 1)) and (MLF1 – GCTTTCCCACGAATAAATAATA [11] – LR) were employed to generate shorter overlapping sequences that allowed the creation of a composite sequence (contig). PCR reactions were carried out in 96 well plates in 12.5 µl reaction volumes containing: 2.5 mM MgCl_2_, 5 pmol of each primer, 20 µM dNTPs, 10 mM Tris HCl (pH 8.3), 50 mM KCl, 10–20 ng (1–2 µl) of genomic DNA, and 1 unit of TaqDNA polymerase (Platinum® Taq DNA Polymerase - Invitrogen) using a thermocycling profile of one cycle of 2 min at 94°C, five cycles of 40 sec at 94°C, 40 sec at 45°C, and 1 min at 72°C, followed by 36 cycles of 40 sec at 94°C, 40 sec at 51°C, and 1 min at 72°C, with a final step of 5 min at 72°C. Products were visualized on a 2% agarose E-Gel® 96-well system (Invitrogen) and samples containing clean single bands were bidirectionally sequenced using BigDye v3.1 on an ABI 3730xl DNA Analyzer (Applied Biosystems). Contigs were made using Sequencher v4.0.5 (Gene Codes) and were subsequently aligned by eye in Bioedit [12]. Sequence divergences were calculated using the K2P distance model [13] and a NJ tree of distances [14] was created to provide a graphic representation of the patterning among-species divergences using MEGA3 [15], and BOLD [16]. Sequence neutrality [Tajima's D - 17] and rates of substitution were calculated with DnaSP v.3 [18]. Sequences and other specimen information are available in the project file “Revision of Malagasy *Anochetus* and *Odontomachus”* in the Published Projects section of the Barcode of Life website (www.barcodinglife.org) with complete collection information for each specimen deposited at www.antweb.org. All sequences from the barcode region have been deposited in Genebank (CO1: EF610629: EF611041, EF999925-EF999945).

**Table 1:** Primers used to generate sequences and molecular tests.

Primer Name	Primer sequence (5′-3′)	Amplicon region	Primer source	Used for sequencing (Y/N)
LepF1	ATTCAACCAATCATAAAGATATTGG	CO1	[56]	Y
LepR1	TAAACTTCTGGATGTCCAAAAAATCA	CO1	[56]	Y
MLepF1	GCTTTCCCACGAATAAATAATA	CO1	[57]	Y
MLepR1	CCTGTTCCAGCTCCATTTT	CO1	[58]	Y
C_ANTMR1D-RonIIdeg_R	GGRGGRTARAYAGTTCATCCWGTWCC	CO1	[Modified from 59]	N
C_ANTMR1D-AMR1deg_R	CAWCCWGTWCCKRMNCCWKCAT	CO1	[Modified from 60]	N
CAS18Fs1	TACACACCGCCCGTCGCTACTA	ITS1	[61]	Y
CAS5p8s1Bd	ATGTGCGTTCRAAATGTCGATGTTCA	ITS1	[Modified from 61]	Y
D2B	GTCGGGTTGCTTGAGAGTGC	28S	[62]	Y
D3Ar	TCCGTGTTTCAAGACGGGTC	28S	[62]	Y
18H3	AGGGTCGATTCCGGAGAGGGAGCCTGAGAA	18S	[63]	Y
185WR	CTTGGCAAATGCTTTCGC	18S	[63]	Y
*wsp* 81F	TGGTCCAATAAGTGATGAAGAAAC	*Wolbachia* surface protein	[20]	Y
*wsp* 691R	AAAAATTAAACGCTACTCCA	*Wolbachia* surface protein	[20]	Y

A composite representation of variation within the CO1 DNA barcode for each of the eight species revised here is presented in Figures 15 and 16. We used the online program Fingerprint [19 - http://evol.mcmaster.ca/fingerprint] to illustrate the heterogeneity at a specific site within the barcode region as a percentage of the vertical line drawn to represent each base pair.

Diagnostic base pairs (or combination of base pairs) for each species within the Malagasy region are presented. This more cladistic interpretation of the DNA barcode data is very sensitive to the number of specimens analyzed – and the fewer specimens incorporated, the greater the likelihood that a rare haplotype is not reflected in the data. We present this method of analysis not to that our coverage of each species is sufficient to reflect the variation within a species, but rather to demonstrate that such an analysis is possible within a group of taxa or region, when there is good representation of the variability within a species. The nucleotide position given refers to the barcode region, and can be compared to their full mitochondrial position by adding 48 (as aligned to the *Bos taurus* complete mitochondrial genome sequence Genbank ref AY676873). The standard IUPAC ambiguity codes are used to denote intra-specific variation.

Complementary genetic analyses. In addition to the CO1 barcode, for some specimens we amplified portions of the rRNA gene regions: 18S, 28S (D2) and ITS1. Within the variable D2 region of 28S, the forward primer corresponds to positions 3549–3568 in *Drosophila melanogaster* reference sequence (Genbank M21017). Within the 18S sequence, the forward primer corresponds to positions 375–406 in *Drosophila melanogaster* reference sequence while the ITS1 sequence was generated using primers where the forward primer corresponds to positions 1822–1843 in *Drosophila melanogaster* reference sequence. Representative sequences have been deposited in Genbank: 18S: EU041960-EU042009; 28S: EU042010-EU042038, EU073708:EU073711; ITS1: EU042039-EU042097, EU073664: EU073707). Primers used to generate these fragments are listed in Table 1. In some cases we utilized a standard PCR diagnostic to test for *Wolbachia* [20]. *Wolbachia* are obligate intracellular endosymbiotic bacteria that cause reproductive incompatibility between infected and uninfected lineages, resulting in an increased proportion of infected maternal lineages that cannot reproduce.

## Results

### Taxonomic synopsis

**Check-List of Malagasy *Anochetus* Species**

*boltoni* sp. nov.
*goodmani* sp. nov.
*grandidieri* Forel, 1891
 = *madecassus* Santschi, 1928
*madagascarensis* Forel, 1887
 = africanus var. friederichsi Forel, 1918
*pattersoni* sp. nov.

**Key to workers and queens of Malagasy *Anochetus***

**Figure 2:**
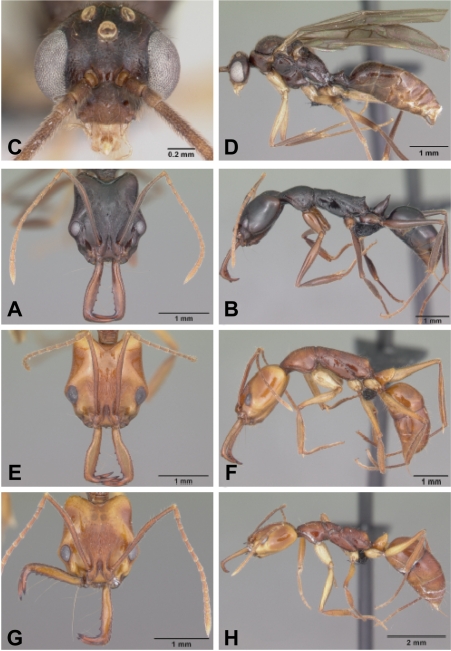
*Anochetus* spp. full face and lateral view. A–B, *boltoni* worker CASENT0104542. C–D, *boltoni* male CASENT0063847. E–F, *goodmani* worker CASENT0104543. G–H, *goodmani* ergatoid queen CASENT0454531.

**Figure 3:**
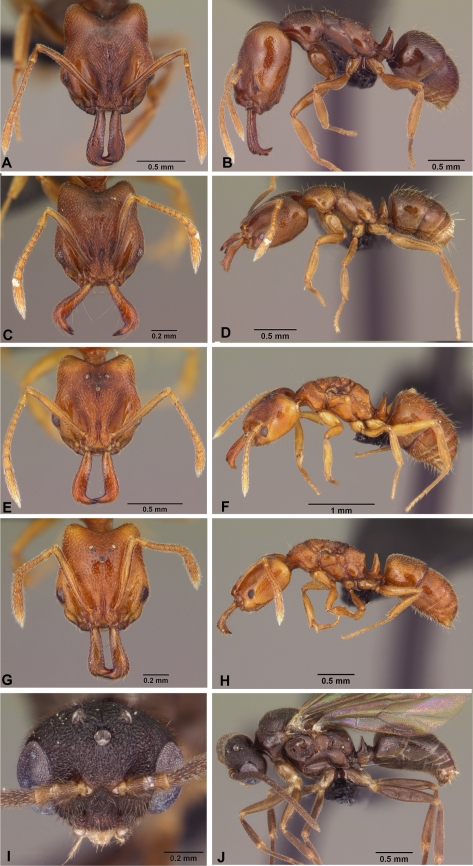
*Anochetus grandidieri* full face and lateral view. A–B, large worker CASENT0497580. C–D, small worker CASENT0033463. E–F, large queen CASENT0041177. G–H, small queen CASENT0498467. I–J, male CASENT0049858.

**Figure 4:**
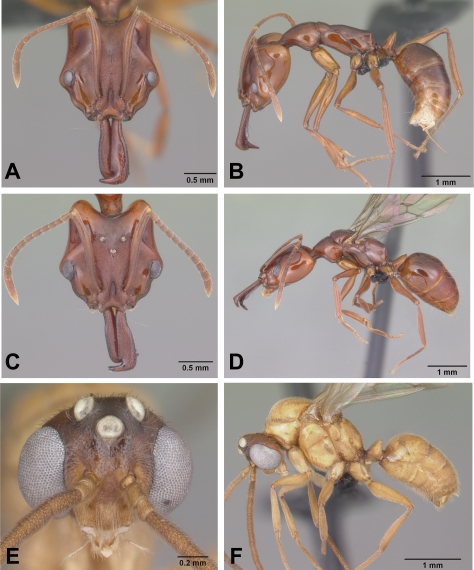
*Anochetus madagascarensis* full face and lateral view. A–B, worker CASENT0104547. C–D, queen CASENT0498419. E–F, male CASENT0049282.

**Figure 5:**
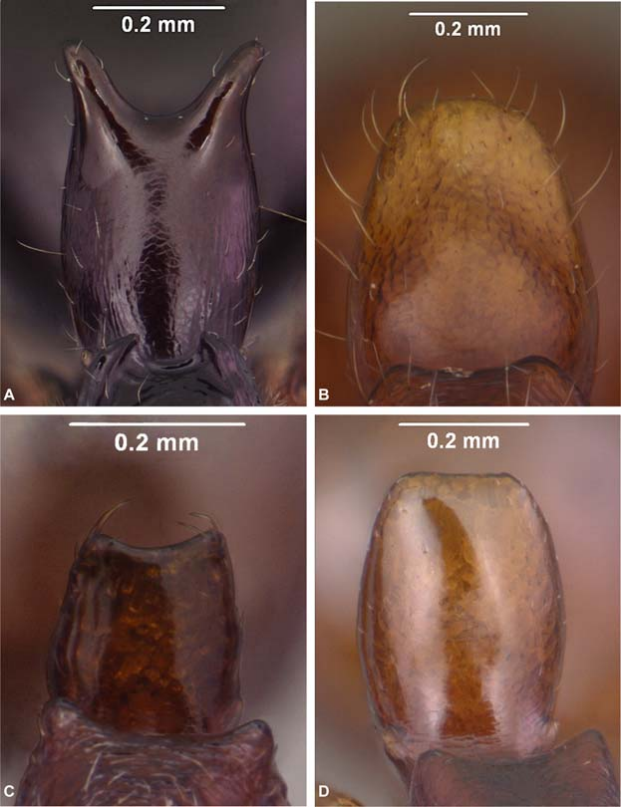
*Anochetus* workers, upper part of petiole from front view. A, *boltoni* CASENT0104542. B, *goodmani* CASENT0104543. C, *grandidieri* (large form) CASENT0497580. D, *madagascarensis* CASENT0498309.

1. Inner mandibular blade without preapical teeth and denticles (Figs 3a, 4a). . .2
  Inner mandibular blade with at least four preapical teeth and denticles (Figs 2a,e). . .4
2. Worker compound eye large, >0.15 mm long. In full face view, antennal scape extends beyond posterior margins of occipital lobe. Dorsal surface of head and mesosoma with or without numerous short setae. . .3
  Worker compound eyes small, <0.15 mm long. In full face view, antennal scape usually fail to reach, and never surpass, posterior margin of occipital lobe. Dorsal surface of head with numerous short setae (Fig. 3a). . .***grandidieri***
3. Dorsal surface of head and mesosoma without numerous short setae (Fig. 3a). Pronotal dorsum glassy smooth. . .***madagascarensis***
  Dorsal surface of head and mesosoma with numerous short setae (Figs 7a,b). Pronotal dorsum with punctures anteriorly and longitudinal ridges posteriorly (Aldabra). . .***pattersoni***
4. Petiolar node as seen from front or rear with apical margin deeply concave, lateral corner forming long spine (Fig. 5a). . .***boltoni***
  Petiolar node as seen from front or rear with apical margin rounded, or slightly flattened, the lateral corner without spine (Fig. 5b). . .***goodmani***

**Key to males of Malagasy *Anochetus*** (males of *goodmani* unknown and not included)

1. Shortest distance between lateral ocellus and margin of compound eye smaller than maximum length of ocellus. Petiolar node as seen from front or rear with lateral corners rounded, without acute spine or sharp tooth. . .2
  Shortest distance between lateral ocellus and margin of compound eye distinctly greater than maximum length of ocellus. Petiolar node as seen from front or rear with lateral corners with acute spine or tooth. . .3
2. Body yellowish brown. Petiolar node as seen from front or rear with apical margin concave. Paramere simple with rounded apex (Fig. 8c). . .***madagascarensis***
  Body dark brown, black. Petiolar node as seen from front or rear with apical margin more or less flat. Paramere constricted apically into a ventrally-directed digitiform lobe (Fig. 8d). . .***pattersoni***
3. Head and mesoscutum with dense reticulate sculpture, opaque, not smooth or shiny. Declivitous surface of propodeum abrupt, about as long as dorsal surface. . .***grandidieri***
  Head and mesoscutum with week sculpture, smooth and shiny areas present. Declivitous surface of propodeum gradually sloping posteriorly, indistinctly delimited from dorsal surface. . .***boltoni***

***Anochetus boltoni*** Fisher **sp. nov.**

urn:lsid:zoobank.org:act:B6C072CF-1CA6-40C7-8396-534E91EF7FBB

Figures: **worker** 2a,b, 5a; **male** 2c,d, 8a; **map** 6a

**Type Material:** *Holotype* worker, MADAGASCAR: Antsiranana, Parc National de Marojejy, Manantenina River, 28.0 km 38° NE Andapa, 8.2 km 333° NNW Manantenina, 14°26′12″S, 049°46′30″E, 450 m, sifted litter, rainforest, 12–15 Nov 2003 (coll. B. L. Fisher et al.), comma collection code: BLF08985 pin code: CASENT0104542 (CASC). *Paratype*. 8 workers with same data as holotype but pins coded, CASENT0487895, CASENT0487896, CASENT0487897, CASENT0006943. (BMNH, MCZ, CAS); CO1 Barcode from paratype collection and coded CASENT0487895-D01

**Worker measurements**: maximum and minimum based on all specimens, n = 20, (holotype): HL 1.80–2.08 (1.95), HW 1.61–1.89 (1.71), CI 87–98 (88), EL 0.33–0.41 (0.36), ML 1.15–1.25 (1.20), MI 59–66 (61), SL 1.83–1.96 (1.84) SI 101–115 (107), WL 2.63–2.89 (2.73), FL 1.97–2.13 (2.03), PW 0.95–1.06 (1.00).

**Male measurements:** maximum and minimum based on n = 2 from Madagascar: HL 0.89–0.91, HW 1.05–1.13, CI 118–125, EL 0.56–0.62, SL 0.24, SI 21–23, WL 2.20–2.24, FL 1.75–1.80

**Worker Diagnosis:** Blade of mandible with five teeth and denticles located along distal two thirds of blade's length. Propodeum with short teeth (Fig. 5a). Dorsolateral margin of petiole with long spine (Fig. 5a). In frontal view, petiolar margin deeply U-shaped. Pilosity, sculpture as in Figures 2a,b.

**Male caste:** Dorsolateral margin of petiole with acute spine.

The species is most similar to *A. goodmani*, but can be easily distinguished by its petiole node with a pair of large apical spines.

**Distribution and biology**. The distribution is limited to collections made between 450 m and 750 m in rainforest in Parc National de Marojejy and 240 m from Ambanitaza near Antalaha (Fig. 4a). It has been collected three times in rotten logs and once in a leaf litter sample. Males have been collected in malaise samples on 20–25 Dec 2004 at 488 m in Parc National de Marojejy

**CO1**. The two populations where collections have been made to date are characterized by a deep divergence within the DNA barcode region (Maximum – 8%) (Fig. 15).

**Diagnostic barcoding loci.** *A. boltoni:* ATCT-42-45 & RTTAR-66-70

**Discussion**: Specimens from Ambanitaza differ notably in shape of propodeal spines and length of spines on petiole from those of the type locality. Though these localities are quite close (40 km apart), these character differences are noticeable, and correspond to significant differences in CO1 (34 base pairs) and ITS1. While specimens from each location were invariant within 18S, there is a 7 bp insertion within ITS1 that is characteristic of the Ambanitaza population which is missing from all specimens from Marojejy. Ultimately, more collections need to be made and evaluated in order to test the hypothesis that these populations represent separate species. One important factor to consider in the testing of that hypothesis is reproductive strategy, which is, to our knowledge, through fission. Though the queen caste is not known, based on overall similarity of workers with *A. goodman*i, it is likely that the queen of *boltoni* is wingless. Queens have never been collected during the 12 month malaise trap sampling even though males were collected. Species that reproduce by fission may show greater geographic differences in morphology and CO1.

**Additional material examined for *Anochetus boltoni:*** In addition to the type material, specimens from 4 additional collecting events from the following three localities were examined in this study. MADAGASCAR: Province **Antsiranana**: Parc National de Marojejy, Manantenina River, 27.6 km 35° NE Andapa; Parc National de Marojejy, Manantenina River, 28.0 km 38° NE Andapa; Forêt Ambanitaza, 26.1 km 347° Antalaha. This material shows greater variation in number of denticles along blade of mandible, ranging from 5–7, compared to the paratypic material.

***Anochetus goodmani*** Fisher **sp. nov.**

urn:lsid:zoobank.org:act:C7D27B95-E1F0-41AC-968C-76BCF3886010

Figures: **worker** 2e,f; **queen** 2g,h; **map** 6a

**Type Material:** *Holotype* worker, MADAGASCAR, Forêt de Binara, 7.5 km 230° SW Daraina, 13°15′18″S, 049°37′00″E, 375 m, 1–4 Dec 2003 (coll. B. L. Fisher et al.), collection code: BLF09638, pin code: CASENT0498309 (CAS). *Paratypes*: 8 workers with same data as holotype but pins coded, CASENT104548, CASENT0498310, CASENT0498311, CASENT0006944, CASENT0006945 (BMNH, MCZ, CAS); CO1 Barcode from paratype collection and coded CASENT0498310-D01.

**Worker measurements**: maximum and minimum based on all specimens, n = 15, (holotype): HL 1.77–2.01 (1.92), HW 1.55–1.81 (1.77), CI 86–92 (92), EL 0.35–0.43 (0.42), ML 1.04–1.15 (1.11), MI 56–66 (58), SL 1.68–1.97 (1.79) SI 101–109 (101), WL 2.52–2.89 (2.66), FL 1.85–2.17 (2.03), PW 0.92–1.06 (1.01).

**Queen (ergatoid) measurements**: maximum and minimum based on n = 5. HL 1.62–1.79, HW 1.49–1.65, CI 91–93, EL 0.37–0.41, ML 0.92–1.02, MI 55–59, SL 1.56–1.71, SI 99–106, WL 2.33–2.55, FL 1.77–1.91, PW 0.88–0.99.

**Figure 6:**
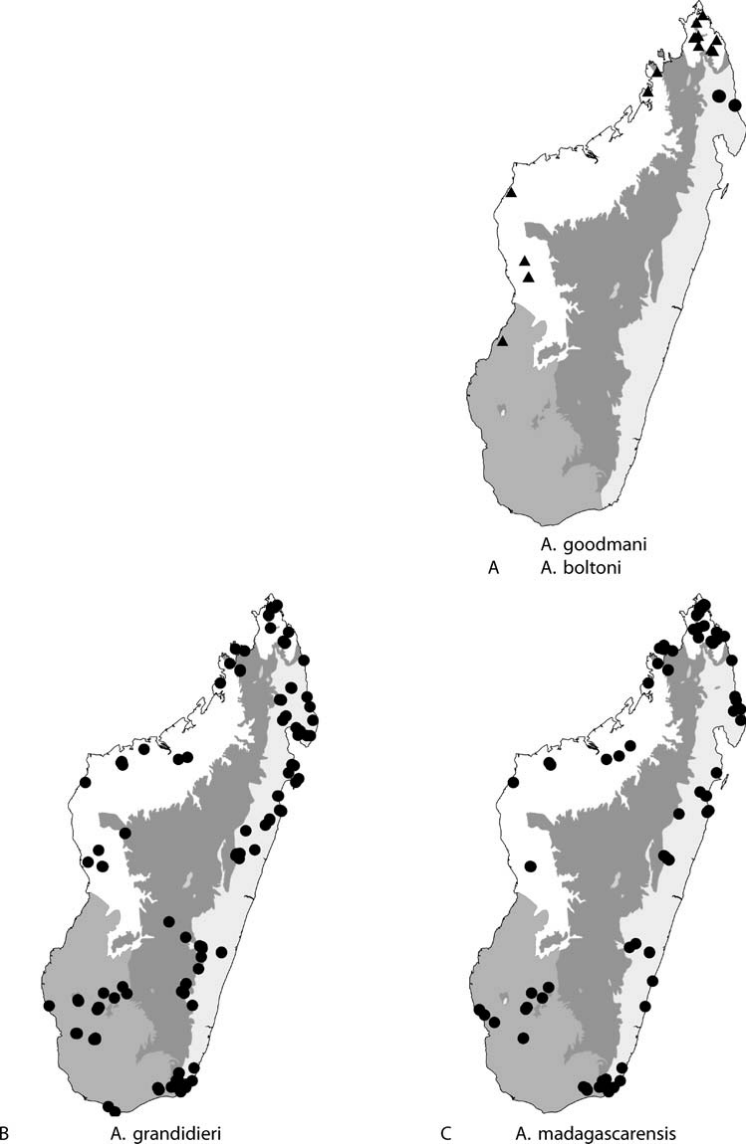
collection localities of *Anochetus* specimens in Madagascar. Map shows major ecoregions: east (light gray): rainforest, central (dark gray): montane forest; west (white): tropical dry forest; southwest (medium gray): desert spiny bush thicket.

**Worker Diagnosis:** Blade of mandible with five teeth and denticles located at the distal half of the blade length. Petiole dorsal margin without spines. In front view, the dorsal petiolar margin flat with lateral margin rounded (Fig. 6b). Pilosity, sculpture as in Figures 2e,f.

The species is most similar to *A. boltoni* but can be easily distinguished by its petiole node without apical spines.

No winged queens are known. Ergatoid queens were collected at six localities. In four of the collections, three ergatoid queens were collected in the same locality. They are very similar in size and shape to workers (Figs 2g,h), and have no ocelli (Fig. 2g). Males are not known.

**Distribution and biology.** *A*. *goodmani* is endemic to Madagascar and is widespread in northern and western parts of the island. It has been collected in dry forest and rainforest as low as 30 m in altitude and also in montane rainforest at the altitude 960 m on Montagne d'Ambre (Fig. 6a), most frequently under stones (12 collections) and sifted litter (7), but also at light (1), beating low vegetation (3), rot pocket (1), in rotten log (6), ground foragers (1), ground nest (9), Malaise trap (1), on low vegetation (1), and pitfall traps (4).

**CO1.** Average intraspecific sequence divergence of 6.37%. There is strong geographic coherence in the divergence patterns (Figs 9, 15, Table 2) with deep divergences occurring between separate regions isolated by habitat and mountains.

**Table 2:** *Anochetus goodmani* within-species pair-wise partitioning of genetic variance for the CO1 DNA barcode. K2P distances are beneath the diagonal and the number of substitutions are above the diagonal.

		Parc National de Kirindy	Parc National Tsingy de Bemaraha	Reserve Speciale de Bemarivo	Antsahabe 550	Antsahabe 550	Antsahabe 550	Ankarana	Ankarana	Ankarana	Antsahabe 550	Antsahabe 550	Antsahabe 550
Parc National de Kirindy	CASENT0015325	**-**	7	17	38	38	39	40	44	43	39	41	39
Parc National Tsingy de Bemaraha	CASENT0432938	0.01101	**-**	16	37	37	38	41	45	44	38	40	38
Réserve Spéciale de Bemarivo	CASENT0022239	0.02704	0.02541	**-**	42	42	43	40	44	43	43	45	43
Forêt d'Antsahabe	CASENT0499942	0.0618	0.06009	0.0686	**-**	0	11	32	37	35	11	13	11
Forêt d'Antsahabe	CASENT0498310	0.0618	0.06009	0.0686	0	**-**	11	32	37	35	11	13	11
Forêt d'Antsahabe	CASENT0076507	0.06349	0.06178	0.0703	0.01737	0.01737	**-**	32	35	35	0	2	0
Réserve Spéciale de Ankarana	CASENT0499020	0.06516	0.06686	0.06515	0.05168	0.05168	0.0517	**-**	7	17	32	34	32
Réserve Spéciale de Ankarana	CASENT0428100	0.07205	0.07374	0.07203	0.06019	0.06019	0.05681	0.01103	**-**	22	35	37	35
Réserve Spéciale de Ankarana	CASENT0505299	0.07032	0.07201	0.07028	0.0567	0.0567	0.05672	0.02704	0.03528	**-**	35	37	35
Forêt d'Antsahabe	CASENT0053814	0.06688	0.06517	0.07373	0.02057	0.02057	0.00313	0.05504	0.06016	0.06008	**-**	2	0
Forêt d'Antsahabe	CASENT0053785	0.06349	0.06178	0.0703	0.01737	0.01737	0	0.0517	0.05681	0.05672	0.00313	**-**	2
Forêt d'Antsahabe	CASENT0053884	0.06349	0.06178	0.0703	0.01737	0.01737	0	0.0517	0.05681	0.05672	0.00313	0	**-**

**Diagnostic barcoding loci**. *A. goodmani:* Y-231 (*madagascarensis* and *grandidieri* A; *boltoni* and *pattersoni* T), W-233 (all others A), RWR-368-370 (others are all ATG), Y-541 (others are all T), R-543 (others are all A), W-546 (others are all T), W-585 (others are all T), M-634 (others are all C). RWCW-42-45 & WTTAG-66-70 (this distinguishes *goodmani* from all (including *boltoni*) except some *madagascarensis*), & GT-83-84 (*madagascarensis* is TA).

**Discussion**. *Anochetus goodmani* is characterized by extreme divergence within the barcode region. To date, sequencing complementary nuclear markers has provided some degree of support for the deepest CO1 divergences (between the north and south-west of Madagascar) as being separate species. Importantly however, ITS1 sequences as divergent have been produced from the same individual (Appendix S1 and Table 3). Although CO1 supports more than one operational unit within *A. goodmani* the hypothesis of cryptic species in relatively isolated environments requires further evidence with less ambiguity.

**Table 3:** Comparison of the utility of various complimentary nuclear markers for species diagnosis in the ponerine ants of the Malagasy.

Taxa	18S	28S	ITS1	Comments
*Anochetus goodmani*	Intra – no variation. Inter – 2 bp from *A. boltoni,* and 3 bp from *O. troglodytes*, *O. coquereli*	Intra – no variation across north Inter – >15 bp divergent from *A. madagascarensis.*	Intra – extreme variation (length and substitution) across range. Some corresponding to deep CO1 splits – provisionally orthologous. However, deep paralogous divergences have been sequenced within single individuals through different amplifications and extractions.	rRNA is, *a priori*, difficult to differentiate orthologous from paraologous. Not as immediately useful as an independent marker without cloning.
*Anochetus boltoni*	Intra – no variation. Inter – 2 bp from *A. goodmani,* 2 bp from *O. troglodytes* and no difference from *O. coquereli.*	N/A	Intraspecific variation of 1% (indels and substitutions) between the two sampled populations.	
*Anochetus madagascarensis*	N/A	Intra – no variation. Inter – >15 bp divergent from *A. goodmani.*	Intra – variation that does NOT reflect CO1 variation.	rRNA is, *a priori*, difficult to differentiate orthologous from paraologous. Not as immediately useful as an independent marker without cloning. -Positive *Wolbachia* test.
*Anochetus grandidieri*	N/A	N/A	Low intraspecific variation that does reflect CO1 geographic variation.	- Positive *Wolbachia* test.
*Anochetus pattersoni*	N/A	N/A	N/A	
*Odontomachus coquereli*	Intra – no variation. Inter – 2 bp from *O. troglodytes.* 2 bp from *A. boltoni*, and 3 bp from *A. goodmani.*	Intra – variation. Large variation at geographically distal ends of distribution. Inter – differentiates between three Malagasy species.	Intraspecific variation that only partially reflects geography and CO1 variation – while some clearly does not. Paralogous and orthologous	
*Odontomachus troglodytes*	Intra – no variation. Inter – 2 bp from *O. coquereli.* 3 bp from *A. boltoni*, and 3 bp from *A. goodmani.*	Intra – some variation that does not correspond to geography or CO1. Inter – does not differentiate between *O. simillimus*	Intraspecific variation that only partially reflects geography and CO1 variation	All specimens tested positive for *Wolbachia.*
*Odontomachus simillimus*	N/A	Intra – no variation. Inter – does not differentiate *O. troglodytes*	N/A	

**Additional material examined for *Anochetus goodmani:*** In addition to the type material, specimens from 56 additional collecting events from the following 18 localities were examined in this study. MADAGASCAR: Province Antsiranana: Montagne des Français, 7.2 km 142° SE Antsiranana; Parc National Montagne d'Ambre; Réserve Spéciale de l'Ankarana, 13.6 km 192° SSW Anivorano Nord; Réserve Spéciale de l'Ankarana, 22.9 km 224° SW Anivorano Nord; Forêt d'Ampondrabe, 26.3 km 10° NNE Daraina; Forêt d' Andavakoera, 21.4 km 75° ENE Ambilobe; 4.6 km 356° N Betsiaka; Forêt d' Antsahabe, 11.4 km 275° W Daraina; Forêt de Binara, 7.5 km 230° SW Daraina; Ampasindava, Forêt d'Ambilanivy, 3.9 km 181° S Ambaliha; Forêt d'Anabohazo, 21.6 km 247° WSW Maromandia; Réserve Spéciale de Bemarivo, 23.8 km 223° SW Besalampy; Parc National Tsingy de Bemaraha, 10.6 km ESE 123° Antsalova; Parc National Tsingy de Bemaraha, 2.5 km 62° ENE Bekopaka, Ankidrodroa River; Parc National Tsingy de Bemaraha, 3.4 km 93° E Bekopaka, Tombeau Vazimba. Province Toliara: Parc National de Kirindy Mite, 16.3 km 127° SE Belo sur Mer.

***Anochetus grandidieri*** Forel

Figures: **worker** 3a–d, 5c; **queen** 3e–h; **male** 3i–j, 8b; **map** 6b

**Type material:**

*Anochetus grandidieri* Forel, 1891: 108 [21]. Lectotype: worker, Madagascar, Forest of the east coast (M. Humblot) (MHNG), **present designation** [examined], AntWeb CASENT0101819. Brown, 1978: 606 [2] (description of worker).

*Anochetus madecassus* Santschi, 1928: 54 [22]. Lectotype: dealate queen, Madagascar, Nossi-Bé (Descarpentries) (NHMB) Lectotype by **present designation** [examined] AntWeb CASENT0101098. Synonymized with *grandidieri* by Brown, 1978: 557 [2].

**Worker measurements:** maximum and minimum based on all specimens, n = 20. HL 0.79–1.19, HW 0.71–1.06, CI 85–95, EL 0.08–0.13, ML 0.33–0.57, MI 41–54, SL 0.57–0.88, SI 78–86, WL 0.87–1.35, FL 0.57–0.90, PW 0.44–0.62.

**Queen measurements:** maximum and minimum based on n = 5. HL 0.88–1.15, HW 0.81–1.07, CI 92–96, EL 0.17–0.23, ML 0.39–0.56, MI 44–49, SL 0.62–0.87, SI 77–81, WL 1.08–1.46. FL 0.68–0.96, PW 0.60–0.78.

**Male measurements:** maximum and minimum based on n = 5 from Madagascar: HL 0.58–0.73, HW 0.78–0.94, CI 129–135, EL 0.37–0.46, SL 0.10–0.15, SI 13–16, WL 1.17–1.52, FL 0.78–1.08

**Worker diagnosis:** Inner blade of mandible without teeth and denticles; apical end of inner blade without a notched semicircular concavity (Fig. 2a). Eyes small (0.05–0.11 mm), projecting dorsolaterally. In full face view, antennal scape usually not reaching, and not surpassing posterior margin of occipital lobe. Dorsal surface of head with numerous short setae. Pilosity and sculpture as in Figures 3 a–d.

**Queens alate:** Very similar to workers, only slightly larger than respective size class (Figs 3e–h). Ergatoid queens not recorded.

Within a single locality, two size classes of workers, queens and males are present in this species, but the differences within a site do not hold up when variation across all sites is included. These differences suggest that two reproductive and developmental pathways can occur in this species. Further work is needed to explore the biotic or abiotic factors that trigger the development of small and large castes.

The species is most similar to *A. madagascarensis* but can be easily distinguished by its small eyes and scape that does not surpass the occipital lobe. *A. madagascarensis* has large eyes (0.24–0.26 mm), and scapes that surpass occipital lobes.

**Distribution and biology**. *A*. *grandidieri* is endemic to Madagascar and is widespread throughout Madagascar in forest and shrubland habitats below 1,550 m elevation (Fig 4b). It has been collected in gallery, dry, littoral, lowland, and montane forest, in desert spiny bush thicket in the southwest, and Uapaca woodland in the central plateau. As in many soil dwelling ants, *A. grandidieri* has reduced eyes (EL/HW 0.11–0.13) and short scapes. *A. grandidieri* is the only *Anochetus* in Madagascar with these soil nesting modifications. The subterranean habitat of this species may allow it to survive in a wide range of habitats in Madagascar from desert to woodland to montane forest. Out of 453 collecting events, *A. grandidieri* was most often recorded in sifted litter (97 collection records), rotten logs (96), and Malaise traps (155).

**CO1.** Shallow intraspecific (average within species sequence divergence of 2.72, SE = 0.048) and deep interspecific divergences (9.4% SE = 0.05) between *A*. *grandidieri* and the other species. Small and large castes had identical DNA barcodes. (Figs 9, 16).

**Diagnostic barcoding loci.** *A grandidieri:* T-273, T-282, T-306, A-312, (shared with one population of *A. goodmani*), A-312, T-333, A-483, T-528 (all 3^rd^ base pair positions).

**Specimens examined for *Anochetus grandidieri:*** Specimens from 456 separate collection events from the following 140 localities were examined. MADAGASCAR: Province **Antsiranana**: Sakalava Beach ; Montagne des Français, 7.2 km 142° SE Antsiranana ; Antsiranana II Pref: Antsahampano S.-Pref: Montagne d'Ambre. Site MD2; Parc National Montagne d'Ambre, 3.6 km 235° SW Joffreville; Réserve Spéciale de l'Ankarana, 13.6 km 192° SSW Anivorano Nord; Forêt d'Ampondrabe, 26.3 km 10° NNE Daraina; Forêt d' Antsahabe, 11.4 km 275° W Daraina; Forêt de Binara, 7.5 km 230° SW Daraina; Forêt de Binara, 9.1 km 233° SW Daraina; Nosy Be, Lokobe Forest; Forêt Ambato, 26.6 km 33° Ambanja; Ambondrobe, 41.1 km 175° Vohemar; Ampasindava, Forêt d'Ambilanivy, 3.9 km 181° S Ambaliha; R.S. Manongarivo, 10.8 km 229° SW Antanambao; R.S. Manongarivo, 12.8 km 228° SW Antanambao; R.S. Manongarivo, 14.5 km 220° SW Antanambao; Forêt d'Anabohazo, 21.6 km 247° WSW Maromandia; Parc National de Marojejy, Manantenina River, 27.6 km 35° NE Andapa, 9.6 km 327° NNW Manantenina; Parc National de Marojejy, Manantenina River, 28.0 km 38° NE Andapa, 8.2 km 333° NNW Manantenina; Parc National Marojejy; Marojejy R.N.I. #12; Forêt Ambanitaza, 26.1 km 347° Antalaha; 9.2 km WSW Befingotra, Rés. Anjanaharibe-Sud; 6.5 km SSW Befingotra, Rés. Anjanaharibe-Sud; 17 km W Andapa, Res. d' Anjanaharibe-Sud; 5 km SW Antalaha; 14 km W Cap Est, Ambato; Fotodriana, Cap Masoala. Province **Mahajanga:** Mahavavy River, 6.2 km 145° SE Mitsinjo; Réserve d'Ankoririka, 10.6 km 13° NE de Tsaramandroso; Ampijoroa National Park, 160 km N Maevatanana, Mahajanga Prov., deciduous forest; Parc National de Namoroka, 17.8 km 329° WNW Vilanandro; Parc National de Namoroka, 16.9 km 317° NW Vilanandro; Parc National de Namoroka, 9.8 km 300° WNW Vilanandro; Réserve Spéciale de Bemarivo, 23.8 km 223° SW Besalampy; Parc National Tsingy de Bemaraha, 10.6 km ESE 123° Antsalova; Forêt de Tsimembo, 8.7 km 336° NNW Soatana; Parc National Tsingy de Bemaraha, 2.5 km 62° ENE Bekopaka, Ankidrodroa River; Parc National Tsingy de Bemaraha, 3.4 km 93° E Bekopaka, Tombeau Vazimba; Province **Toamasina**: Montagne d'Anjanaharibe, 19.5 km 27° NNE Ambinanitelo; Montagne d'Anjanaharibe, 18.0 km 21° NNE Ambinanitelo; Montagne d'Akirindro 7.6 km 341° NNW Ambinanitelo; 19 km ESE Maroantsetra; 6.9 km NE Ambanizana, Ambohitsitondroina; Ambanizana, Parc National Masoala; 5.3 km SSE Ambanizana, Andranobe; 6.3 km S Ambanizana, Andranobe; 1 km W Andampibe, Cap Masoala; Parc National Mananara-Nord, 7.1 km 261° Antanambe; Forêt d'Analava Mandrisy, 5.9 km 195° Antanambe; Res. Ambodiriana, 4.8 km 306°Manompana, along Manompana River; Ile Sainte Marie, Forêt Ambohidena, 22.8 km 44° Ambodifotatra; Ile Sainte Marie, Forêt Kalalao, 9.9 km 34° Ambodifotatra; Parcelle E3 Tampolo; S.F. Tampolo, 10 km NNE Fenoarivo Atn.; Bridge at Onibi, NW of Mahavelona; Mahavelona (Foulpointe); 2.1 km 315° Mahavelona; Foulpointe; Reserve Betampona, Camp Vohitsivalana, 37.1 km 338° Toamasina; Reserve Betampona, Camp Rendrirendry 34.1 km 332° Toamasina; F.C. Sandranantitra; F.C. Didy; F.C. Andriantantely; P.N. Mantadia; Analamay; Forêt Ambatovy, 14.3 km 57° Moramanga; Torotorofotsy; Andasibe National Park, botanic garden near entrance, West of ANGAP office; Res. Analamazaotra, Parc National, Andasibe; **Fianarantsoa:** Forêt d'Atsirakambiaty, 7.6 km 285° WNW Itremo; Ranomafana Nat. Park, Miaranony Forest; Vohiparara broken bridge, Fianarantsoa Prov.; Parc National de Ranomafana, Sahamalaotra River, 6.6 km 310° NW Ranomafana; Parc Nationale Ranomafana: Talatakely; 3 km W Ranomafana, nr. Ifandiana; research cabin at Talatakely, Ranomafana National Park; radio tower, Ranomafana National Park, Fianarantsoa Prov.; Namorona River at footbridge, Ranomafana National Park; Ranomafana National Park, Tavolo tree; Belle Vue trail, Ranomafana National Park, Fianarantsoa Prov.; 7 km W Ranomafana; Vatoharanana; Parc National de Ranomafana, Vatoharanana River, 4.1 km 231° SW Ranomafana; P.N. Ranomafana, Vatoharanana-Ankovoka; 8 km E Kianjavato, Vatovavy Forest; 7.6 km 122° Kianjavato, Forêt Classée Vatovavy; 2 km W Andrambovato, along river Tatamaly; Forêt d'Ambalagoavy Nord, Ikongo, Ambatombe; 45 km S. Ambalavao; 45 km S Ambalavao; 43 km S Ambalavao, Rés. Andringitra; Parc National d'Isalo, Ambovo Springs, 29.3 km 4° N Ranohira; 8.0 km NE Ivohibe; 9.0 km NE Ivohibe; R.S. Ivohibe, 7.5 km ENE Ivohibe; Parc National d'Isalo, 9.1 km 354° N Ranohira; Forêt d'Analalava, 29.6 km 280° W Ranohira; Forêt de Vevembe, 66.6 km 293° Farafangana; Province **Toliara:** Réserve Spéciale d'Ambohijanahary, Forêt d'Ankazotsihitafototra, 34.6 km 314° NW Ambaravaranala; Réserve Spéciale d'Ambohijanahary, Forêt d'Ankazotsihitafototra, 35.2 km 312° NW Ambaravaranala; Vohibasia Forest, 59 km NE Sakaraha; southern Isoky-Vohimena Forest, 59 km NE Sakaraha; Forêt Classée d'Analavelona, 33.2 km 344° NNW Mahaboboka; Forêt Classée d'Analavelona, 29.2 km 343° NNW Mahaboboka; Forêt Classée d'Analavelona, 29.4 km 343° NNW Mahaboboka; Forêt de Tsinjoriaky, 6.2 km 84° E Tsifota; Parc National de Zombitse, 19.8 km 84° E Sakaraha; Parc National de Zombitse, 17.7 km 98° E Sakaraha; 15 km E Sakaraha; Forêt de Mite, 20.7 km 29° WNW Tongobory; Sept Lacs; Beza-Mahafaly, 27 km E Betioky; Ehazoara Canyon, 26 km E Betioky; 70.7 km NNE Tolanaro, Mahermano Mt.; 11 km NW Enakara, Rés. Andohahela; 10 km NW Enakara, Rés. Andohahela; Rés. Andohahela, 6 km SSW Eminiminy; Parc National d'Andohahela, Col du Sedro, 3.8 km 113° ESE Mahamavo, 37.6 km 341° NNW Tolagnaro; Parc National d'Andohahela, Manampanihy River, 5.4 km 113° ESE Mahamavo, 36.7 km 343° NNW Tolagnaro; 2.7 km WNW 302° Ste. Luce; 9.2 km N Tolanaro, Ilapany Mt.; 29.5 km WNW Tolanaro, Vasiha Mt.; Parc National d'Andohahela, Forêt d'Ambohibory, 1.7 km 61° ENE Tsimelahy, 36.1 km 308° NW Tolagnaro; Mandena, 8.4 km NNE 30° Tolagnaro; Réserve Privé Berenty, Forêt de Bealoka, Mandraré River, 14.6 km 329° NNW Amboasary; Réserve Privé Berenty, Forêt de Malaza, Mandraré River, 8.6 km 314° NW Amboasary; Réserve Berenty; Forêt de Petriky, 12.5 km W 272° Tolagnaro; 4.4 km 148° SSE Lavanono; Réserve Spéciale de Cap Sainte Marie, 14.9 km 261° W Marovato; near road, Zombitse National Park, Tulear Prov.; near ANGAP office, Zombitse National Park, Tulear Prov.; Parcel I, Beza Mahafaly Reserve, near research station, Tulear Province; Tsimelahy - Parcel II, Andohahela National Park, transition forest, Tulear Province.

***Anochetus madagascarensis*** Forel

Figures: **worker** 1a, 4a,b, 5d; **queen** 4c,d; **male** 4e,f, 8b; **map** 6c

**Type material:**

*Anochetus africanus madagascarensis* Forel, 1887: 382 [23]. Lectotype: worker, Madagascar, Tamatave Province, Ivondro, (Dr. Conrad Keller) (MHNG) **present designation** [examined] AntWeb CASENT0101574. Raised to species by Brown, 1978: 557 [2].

*Anochetus africanus friederichsi* Forel 1918: 155 [24]. Lectotype: worker, Madagascar, Tamatave Province, Prune Island (Nosy Alanana) (Friederichs) (MHNG), **present designation** [examined] AntWeb CASENT010165. Synonymized with *madagascarensis* by Brown, 1978: 557 [2].

**Worker measurements:** maximum and minimum based on n = 20. HL 1.35–1.68, HW 1.19–1.53, CI 87–94, EL 0.23–0.28, ML 0.73–0.93, MI 53–57, SL 1.11–1.41, SI 89–95, WL 1.60–2.02, FL 1.13–1.54, PW 0.63–0.80.

**Queen measurements:** maximum and minimum based on n = 5. HL 1.52–1.66, HW 1.48–1.55, CI 92–97, EL 0.32–0.36, ML 0.81–0.89, MI 53–55, SL 1.26–1.39, SI 85–91, WL 1.99–2.22. FL 1.35–1.49, PW 0.84–0.92.

**Male measurements:** maximum and minimum based on n = 5 from Madagascar: HL 0.85–1.89, HW 1.07–1.20, CI 122–135, EL 0.63–0.69, SL 0.20–0.22, SI 18–21, WL 1.90–1.98, FL 1.35–1.47

**Worker Diagnosis:** Inner blade of mandible without teeth and denticles; apical end of inner blade with notched semicircular concavity (Fig. 1a). Eyes large (0.24–0.26 mm), projecting dorsally. In full face view, antennal scape extends beyond posterior margin of occipital lobe. Dorsal surface of head asetose. Pilosity and sculpture as in Figures 4a,b.

**Queens alate:** Very similar to worker and only slightly larger (Figs 4c,d). Queens of only one size. Ergatoid queens not recorded.

**Males:** Males light yellowish brown in color and with large projecting ocelli on vertex (Figs 4e,f). Males have been collected in Malaise traps in every month of the year and males have been noted to swarm and fly at dusk and early evening.

The species is most similar to *A. grandidieri* but can be easily distinguished by its large eyes (0.24–0.26 mm), and scapes that surpass occipital lobes.

**Distribution and biology.** *A*. *madagascarensis* is widespread throughout Madagascar in forest or shrubland habitats below 1100 m elevation and is also known from the Comoros. Forel's (1912:159) record of a male “*Anochetus* sp.? *africanus* var. *madagascariensis* Forel” from Seychelles, Mahé, has not been seen and confirmed. This record most likely refers to *pattersoni*. In Madagascar, *madagascarensis* is widespread and has been collected in gallery, dry, littoral, lowland, and montane forests, and in desert spiny bush thicket in the southwest of Madagascar. The longer scapes and larger eyes of *A. madagascarensis* compared to *A. grandidieri,* correlate with nesting and foraging above the soil layer. The species was most often recorded nesting in rotten logs (99 collection records) followed by sifted litter (41). In addition, it was collected from dead twigs above ground (1), rot pockets (2), ground foragers (20), ground nests (6), Malaise trap (14), on low vegetation (2), and pitfall traps (4).

**CO1.** Shallow intraspecific and deep interspecific divergences between *A*. *madagascarensis* and the other species. Average within species sequence divergence of 1.67% (SE = 0.055) (Fig. 16).

**Diagnostic barcoding loci.** *A. madagascarensis*: A-21, T-423 (shared with one *A. goodmani* population), T-132 (shared with one *A. grandidieri* population), T-83, A-84, T-93, T, 138, C-306, T-513, A-595

**Specimens examined for *Anochetus madagascarensis*:**

Specimens from 326 separate collection events from the following 129 localities were examined.

COMORES: **Mayotte Island**: Majimbini; Coconi DAF campus; Poroani; Riv. Kouale nr. Caserne; Convalescence; Dziani Karihani; Tsingoni; Mt. Choungui; Mt. Combani; Coconi, SDA (service du develppement agricole); Mt. Benara; Sazile; MADAGASCAR**: Antsiranana**: Nosy Be 5 km SE Marodokana; ridge behind Sambava, Q-37; Antalaha 18 km North; Nossi bé;; 68 km SW of Sambava; Ambohitsara, 10 km SW Antalaha; 2 km W Antalaha; Soavinandriana; 2 km S Antalaha; Orangea, 3 km E Ramena [near fort]; Forêt d'Orangea, 3.6 km 128° SE Remena; Sakalava Beach; 1 km W Sakalava Beach; 3 km W Sakalava Beach; Montagne des Français, 7.2 km 142° SE Antsiranana; Montaigne Francais; 7 km N Joffreville; Réserve Spéciale d'Ambre, 3.5 km 235° SW Sakaramy; Parc National Montagne d'Ambre; Parc National Montagne d'Ambre; Parc National Montagne d'Ambre [Petit Lac road]; Parc National Montagne d'Ambre, 3.6 km 235° SW Joffreville; Rés. Analamerana, 16.7 km 123° Anivorano-Nord; Réserve Spéciale de l'Ankarana, 13.6 km 192° SSW Anivorano Nord; Ankarana; Res. Ankarana; Réserve Spéciale de l'Ankarana, 22.9 km 224° SW Anivorano Nord; Forêt d'Ampondrabe, 26.3 km 10° NNE Daraina; Forêt d'Analabe, 30.0 km 72° ENE Daraina; Forêt d' Andavakoera, 21.4 km 75° ENE Ambilobe; 4.6 km 356° N Betsiaka; Forêt de Bekaraoka, 6.8 km 60° ENE Daraina; Forêt d' Antsahabe, 11.4 km 275° W Daraina; Forêt de Binara, 7.5 km 230° SW Daraina; Forêt de Binara, 9.1 km 233° SW Daraina; Nosy Be, Airport; Nosy Be, 5 km Marodokana; Nosy be, Ambatoloaka; Nosy Be, Lokobe Forest; Nosy Be, 4 km ESE Andoany ( = Hellville); Nosy Be, Réserve Naturelle Intégrale de Lokobe, 6.3 km 112° ESE Hellville; Forêt Ambato, 26.6 km 33° Ambanja; Ambondrobe, 41.1 km 175° Vohemar; Ampasindava, Forêt d'Ambilanivy, 3.9 km 181° S Ambaliha; R.S. Manongarivo, 10.8 km 229° SW Antanambao; R.S. Manongarivo, 12.8 km 228° SW Antanambao; Forêt d'Anabohazo, 21.6 km 247° WSW Maromandia; Forêt Ambohibato, 27.2 km 349° Antalaha; Forêt Ambanitaza, 26.1 km 347° Antalaha; 18 km N Antalaha, Ampahana; 5 km S+5 km W Antalaha; Antalaha, 12 km S; Marofinaritra; 14 km W Cap Est, Ambato; **Mahajanga**: Forêt Ambohimanga, 26.1 km 314° Mampikony; Parc National d'Ankarafantsika, Forêt de Tsimaloto, 18.3 km 46° NE de Tsaramandroso; Ampijoroa National Park, 160 km N Maevatanana, Mahajanga Prov., deciduous forest; Parc National de Namoroka, 16.9 km 317° NW Vilanandro; Parc National de Namoroka, 9.8 km 300° WNW Vilanandro; Réserve Spéciale de Bemarivo, 23.8 km 223° SW Besalampy; Parc National Tsingy de Bemaraha, 2.5 km 62° ENE Bekopaka, Ankidrodroa River; Parc National Tsingy de Bemaraha, 3.4 km 93° E Bekopaka, Tombeau Vazimba; **Toamasina**: Ivondro p. Tamatavé; Ilât Prune bei Tamatave; Tamatave; Res. Betampona, Ambodiriana 45 km NW Toamasina; Res. Ambodiriana, 4.8 km 306°Manompana, along Manompana river; Parcell K9 Tampolo; S.F. Tampolo, 10 km NNE Fenoarivo Atn.; Parcelle E3 Tampolo; Mahavelona (Foulpointe); Analalava, 7.0 km 255° Mahavelona; Manakambahiny Atsinanana; Forêt Ambatovy, 14.3 km 57° Moramanga; Torotorofotsy; Andasibe National Park, botanic garden near entrance, West of ANGAP office; 7 km SE Andasibe National Park Headquarters; **Fianarantsoa**: Riv: Morongolo Aff de Rongaronga; Local: Antanandava PK 285 RN2; Nat. Pk. Ranomafana, Miaranony Forest; Ranomafana Nat. Park; Ranomafana Nat. Park, Vohiparara, Hotel; 8 km NE Kianjavato, Vatovavy forest; Nat. Pk.Ranomafana, Miaranony Forest; Ranomafana Nat. Park, Tsarahomanana; 7 km W Ranomafana; 8 km E Kianjavato, Vatovavy Forest; 7.6 km 122° Kianjavato, Forêt Classée Vatovavy; Manakara; Parc National d'Isalo, Sahanafa River, 29.2 km 351° N Ranohira; Forêt d'Analalava, 29.6 km 280° W Ranohira; Farafangana; 29.5 km WNW Tolanaro, Vasiha Mt.; **Toliara**: Andohahela, Parcel #1 versante E.; 29 km NNW Ranohira, Isalo N.P.; Vohibasia Forest, 59 km NE Sakaraha; near road, Zombitse National Park, Tulear Prov.; near ANGAP office, Zombitse National Park, Tulear Prov.; Mikea Forest, deciduous dry forest, Tulear Province; Mikea Forest, spiny forest, Tulear Province; Ranobe; Fiherenana; Beza-Mahafaly, 27 km E Betioky; Beza-Mahafaly, Parcel 1; 70.7 km NNE Tolanaro, Mahermano Mt.; Rés. Andohahela, 6 km SSW Eminiminy; 2.7 km WNW 302° Ste. Luce; Andohahela; Réserve Privé Berenty, Forêt d'Anjapolo, 21.4 km 325° NW Amboasary; Tsimelahy - Parcel II, Andohahela National Park, transition forest, Tulear Province; Mandena, 8.4 km NNE 30° Tolagnaro; Réserve Privé Berenty, Forêt de Bealoka, Mandraré River, 14.6 km 329° NNW Amboasary; Réserve Privé Berenty, Forêt de Malaza, Mandraré River, 8.6 km 314° NW Amboasary; Réserve Berenty; Res. Berenty; Forêt de Petriky, 12.5 km W 272° Tolagnaro.

***Anochetus pattersoni*** Fisher **sp. nov.**

urn:lsid:zoobank.org:act:A1B9370B-2286-41D0-8E28-335C3514A76A

Figures: **worker** 7a–d; **queen** 7e,f; **male** 7g,h, 8d

**Type Material:** *Holotype*: worker, Seychelles Aldabra Group, Picard Island, in old “Settlement” 09°23′34″S 046°12′14″E 5 m, mostly *Casuarina* with coco palms, exotic vegetation, found after dark on concrete slab in abandoned settlement 19-Dec-05 (coll. S.M.Goodman) collection code: SMG14998 CASENT0068352 1w (CASC). CO1 barcode from same collection as holotype and labeled CASENT0068352-D01

**Worker measurements**: maximum and minimum based on all specimens, n = 8, (holotype): HL 1.32–1.40 (1.40), HW 1.25–1.31 (1.31), CI 93–95 (94), EL 0.20–0.26 (0.23), ML 0.67–0.72 (0.72), MI 50–51 (51), SL 1.07–1.15 (1.15) SI 85–88 (88), WL 1.62–1.79 (1.78), FL 1.11–1.20 (1.19), PW 0.70–0.76 (0.74).

**Queen measurements**: maximum and minimum based on n = 1. HL 1.31, HW 1.29, CI 99, EL 0.30, ML 0.64, MI 49, SL 1.05, SI 81, WL 1.81, FL 1.15, PW 0.79.

**Male measurements:** maximum and minimum based on n = 2 from Madagascar: HL 0.86–0.87, HW 1.07–1.10, CI 124–126, EL 0.65–0.67, SL 0.18, SI 17, WL 1.72–1.77, FL 1.21–1.26

**Figure 7:**
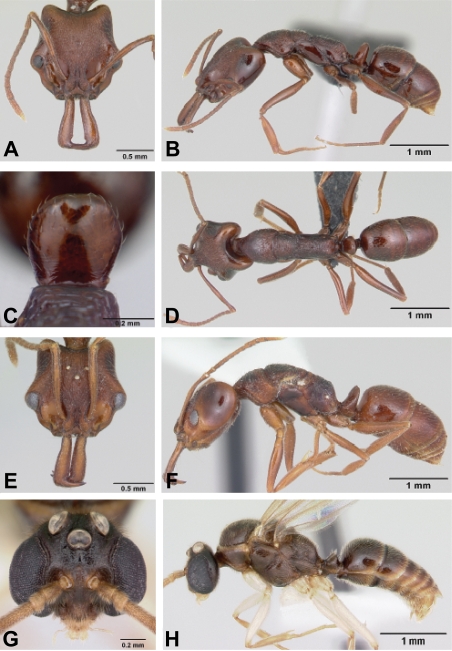
*Anochetus pattersoni*. A–D Worker holotype CASENT0102280 full face, lateral view, upper part of petiole from rear view, dorsal view. E–F, queen paratype CASENT0103343 full face and lateral view. G–H, male CASENT0172617 full face and lateral view.

**Worker Diagnosis**: Dorsal margin of petiole node concave medially (not visible in figures of the workers but easily seen in the queen in Figure 7f.) Anterior portion of pronotal dorsum lightly sculptured compared to posterior portion of pronotum. Propodeal dorsum and angle transversely coarsely rugose, declivitous face below angle with transverse striae, with sculpture thinning near base of face; propodeum angulate in lateral view. Petiole scale broad; anterior half of first gastral tergum smooth and shiny with only fine punctures at base of setae. This species is most similar to the *graeffei* a widespread species across the Indo-Pacific, but differs from the latter by the pattern of sculpture on the mesosomal dorsum, shape the petiole (concave), broader petiole node as seen in lateral view, and its much larger size (HL+ML 1.99–2.12 mm in *pattersoni*, HL+ML<1.75 mm in *graeffei*).

**Distribution and biology.** This species is limited to the Aldabra group islands with most collections from Isle Picard. References and records to *Anochetus madagascarensis* [e.g. Forel 25:159] most likely refer to this species. No other species of *Anochetus* have been recorded from the Seychelles. Males have been collected in Malaise traps, and a queen with clear wing scares.

**Diagnostic barcoding loci:** *A. pattersoni*: G-183, G-264, A-399, A-489, A-505, A-552.

**Additional material examined for** ***Anochetus pattersoni:*** In addition to the type material, specimens from the following localities were examined in this study. **Seychelles**: **Aldabra Group**: South Island (Grand Terre), Dune Patates 5-Jun-74 (Coll: V. Spaull) CASENT0102280 3w (BMNH); Isle Picard 12–25 Mar-85 (Col: P.Mundel) CASENT0103343 1dQ, CASENT0103344 1w (CASC), MCZ.3680w 1w (MCZC); Ile Picard Settlement, 11; (ANIC32-015992) 1-Nov-68 (coll: W.F.Humphreys) CASENT0172374 1w (ANIC); Ile Polymnie, Anse Cedres, 155; (ANIC32-015991) 1-Nov-68 (coll: W.F.Humphreys) CASENT0172375 1w (ANIC); Cosmoledo, Menai 17-Dec-05 (col: J.Gerlach) CASENT0172609 1w (LACM); Grande Terre, Aldabra 15-Dec-05; (coll: J.Gerlach) CASENT0172610 1w (LACM); Aldabra Islands, Picard 22–29 Sep-05 ex malaise trap 6 m (coll: K.Mach & O.Maurel) CASENT0172611 1 m (LACM); Aldabra Islands, Picard 22–26 May-05 (coll: K.Mach & O.Maurel) CASENT0172617 1 m (LACM).

**Check-List of Malagasy *Odontomachus* Species**

*coquereli* Roger, 1861
 = coquereli minor Emery, 1899
*troglodytes* Santschi, 1914
 = haematodus stanleyi Wheeler 1922
*simillimus* Smith 1858
 = haematoda breviceps, Crawley 1915
 = haematodes fuscipennis Forel 1913
 = *pallidicornis* Smith, F. 1860

**Key to workers and queens of Malagasy *Odontomachus*** (modified from Brown [1:117]

1. Head narrow behind eyes; mandible with long, acute apical and preapical teeth; vertex of head coarsely, transversely striate. . .***coquereli***
  Head only slightly narrower across vertex than across eyes, with distinct extraocular furrows and temporal ridges; apical and preapical teeth of mandible short and blunt; vertex finely striate longitudinally, diverging behind. . .2
2. Metasternal process acute, forming paired, slender spines, often unequal in length (Fig. 13a). Petiole spine notably bent posteriorly at base. . .***troglodytes***
  Metasternal process low, rounded (Fig. 13b). Petiole spine slightly curved posteriorly, comma but not noticeably bent posteriorly at base of spine. . .***simillimus***

**Key to males of Malagasy *Odontomachus***

1. Shortest distance between lateral ocellus and margin of compound eye smaller than maximum length of ocellus. Antenna with suberect setae; declivitous surface of propodeum without distinct rugae (Madagascar). . .***coquereli***
  Shortest distance between lateral ocellus and margin of compound eye distinctly greater than maximum length of ocellus. Antenna with very short appressed to decumbent setae; declivitous surface of propodeum with distinct rugae directed towards margins. . .2
2. Body brownish yellow. Tarsal claw with small subapical tooth (Madagascar). . .***troglodytes***
  Body blackish or brown. Tarsal claw without subapical tooth (Seychelles). . .***simillimus***

***Odontomachus coquereli*** Roger

Figures: **worker** 1b, 10a,b, 13c; **queen** 10c,d; **male** 11a,b,e; **map** 14a

**Type material:**

*Odontomachus coquereli* Roger, 1861: 30 [26]. Lectotype: worker, Madagascar (Coquerel) (ZMHB), **present designation** [examined] AntWeb CASENT0104549.

*Odontomachus coquereli minor* Emery 1899: 273 [27]. Lectotype; worker, Madagascar, Baie d' Antongil (Mocquerys) (MSNG), **present designation** [examined] AntWeb CASENT0102021. Synonymized with *coquereli* by Brown, 1978: 557 [2].

**Worker measurements:** maximum and minimum based on n = 45 from Madagascar: HL 2.69–3.27, HW (across vertex) 1.26–1.77, HW (across upper eye margin) 1.54–2.02, CI 57–67, EL 0.46–0.55, ML 1.76–2.16, MI 61–68, SL 3.04–3.96, SI 164–207, WL 4.18–5.11. FL 3.32–4.68, PW 1.11–1.53.

**Queen measurements:** maximum and minimum based on n = 5 from Madagascar: HL 2.81–2.94, HW (across vertex) 1.39–1.55, HW (across upper eye margin) 1.83–1.98, CI 62–71, EL 0.45–0.55, ML 1.66–1.81, MI 59–62, SL 3.07–3.29, SI 155–179, WL 4.35–4.56, FL 3.60–3.84, PW 1.28–1.43. Preapical teeth count 7–10.

**Male measurements:** maximum and minimum based on n = 5 from Madagascar: HL 1.11–1.22, HW 1.41–1.57, CI 128–134, EL 0.78–0.90, SL 0.30–0.38, SI 21–23, WL 3.38–3.85, FL 2.90–3.16.

**Worker Diagnosis:** Workers of this species can be easily distinguished from *troglodytes* by their larger size, mandible with long, acute apical and preapical teeth and lack of extraocular furrows and temporal ridges on vertex. Brown [2] provides a description and additional references.

**Distribution and biology.** *O*. *coquereli* is endemic to Madagascar and is restricted to eastern and northern montane rainforest, lowland rainforest, and littoral forest from 10 to 1325 m (Fig. 10a). It is most abundant at mid-elevations in the northeast such as in Marojejy National Park. Nests of *O. coquereli* are most commonly found in rotten logs and consist of small colonies. Queens of *coquereli* are wingless and very similar to workers; colonies reproduce by fission [28]. Males are collected in Malaise traps and yellow pan traps. Workers forage on the ground day and night. A few times BLF has seen solitary foragers high up on trunks and branches of large trees. It is not clear if they are foraging for plant or insect liquids up in the canopy.

**Figure 8:**
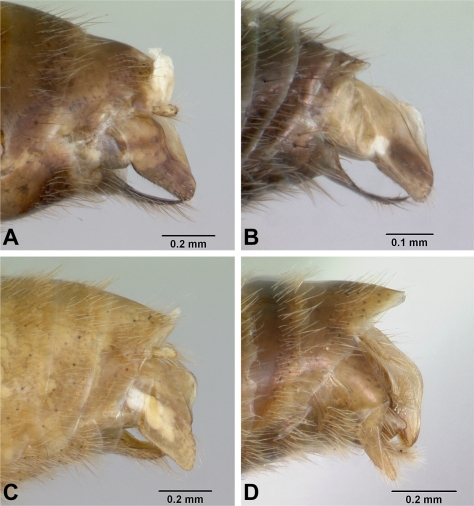
*Anochetus* males, terminalia, lateral view. A, *boltoni* CASENT0063847. B, *grandidieri* CASENT0080660. B, *madagascarensis* CASENT0063421. D, *pattersoni* CASENT0172617.

**Figure 9:**
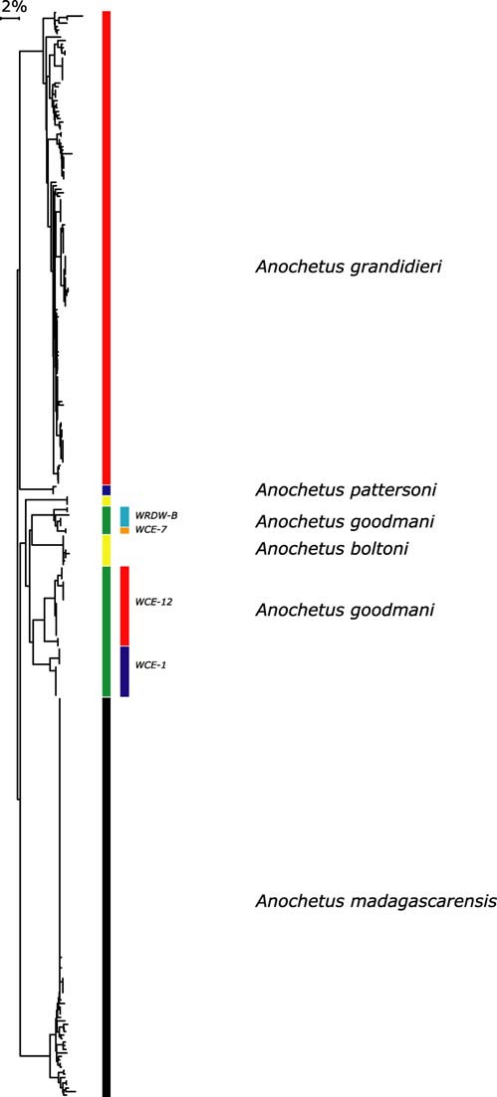
NJ tree of K2P for five species of *Anochetus* in Madagascar, Comoros and Aldabra (all specimens with >500 bp). Deep divergences evident between *madagascarensis, grandidieri,* and *goodmani* are evident. Deep divergences within *A. goodmani* are evident (In this tree, *A. boltoni* falls within *goodmani*). The rightmost column of colors differentiate which biogeographical groupings of Wilmé *et al*. [29] these populations fall. WCE-1 = Binara, Antsahabe. WCE-12 = Andavakoera, Ankarana. WCE-7 = Kirindy Mite. WRDW-B = Vazimba, Androngonibe, Andranopasazy.

**Figure 10:**
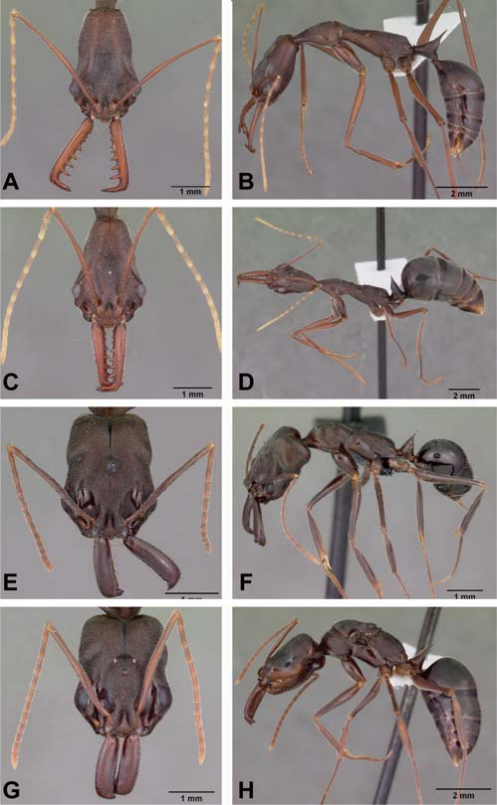
*Odontomachus* spp. full face and lateral view. A–B, *coquereli* worker CASENT0009409. C–D, *coquereli* ergatoid queen CASENT 0104947. E–F, *troglodytes* worker CASNET0047308. G–H, *troglodytes* dealate queen CASENT0100313.

There is notable geographic variation in shape of petiole, sculpture and number of preapical teeth. Preapical teeth and denticles range from 7–12. Occasionally, adjacent teeth may be fused at base to form a single bidententate tooth. However, there is no consistent concordant pattern to this variation. Molecular data are also extremely variable – suggesting that these isolated populations have long been separated. Rather than describing these populations as distinct species, we leave them here as a single species – a hypothesis that can be tested in the future with subsequent experiments in both the field and lab.

**CO1:** The barcode region is extremely variable (Fig. 16) – there is evident isolation by distance which is largely concordant with the biogeographic regions proposed by Wilme *et al*. [29].

**Diagnostic barcoding loci.** *O. coquereli*: T-96, C-196, T-211, T-280, A-283.

**Discussion:** *Odontomachus coquereli* from Madagascar, the only species in the genus where winged queens have never been found. Molet *et al*. [28] investigated the Marojejy population of *O*. *coquereli*, and based on demography, morphometry, allometry and ovarian dissections demonstrated that the winged queen caste has been replaced by a wingless reproductive caste and that the strategy of colonial reproduction is fission. A single wingless reproductive (ergatoid) was found in each colony. The smallest colonies consisted of at least 5 workers and the largest colonies never exceeded 40 workers, indicating a threshold size at which a colony divides in two daughter colonies. In contrast, *O. troglodytes* reproduces by non-claustral independent foundation and colonies can reach 1300 workers [30]. As in *A. goodmani* and *A. boltoni*, the other species without winged queens – there are deep CO1 divergences between different collection localities.

**Specimens examined for *Odontomachus coquereli***:

Specimens from 134 separate collection events from the following 57 localities were examined. MADAGASCAR: Province **Antsiranana**: Forêt de Binara, 9.4 km 235° SW Daraina; R.S. Manongarivo, 12.8 km 228° SW Antanambao; R.S. Manongarivo, 14.5 km 220° SW Antanambao; RNI Marojejy, 8 km NW Manantenina; Parc National de Marojejy, Manantenina River, 27.6 km 35° NE Andapa; Parc National de Marojejy, Manantenina River, 28.0 km 38° NE Andapa; Parc National de Marojejy, Antranohofa, 26.6 km 31° NNE Andapa; Forêt Ambanitaza, 26.1 km 347° Antalaha; Rés. Anjanaharibe-Sud, 6.5 km SSW Befingotra,; Res. D' Anjanaharibe-Sud, 17 km W Andapa; Province **Toamasina**: 6.9 km NE Ambanizana, Ambohitsitondroina; Montagne d'Anjanaharibe, 19.5 km 27° NNE Ambinanitelo; Montagne d'Anjanaharibe, 18.0 km 21° NNE Ambinanitelo; Montagne d'Akirindro 7.6 km 341° NNW Ambinanitelo; Parc National Masoala, Ambanizana, ; 5.3 km SSE Ambanizana, Andranobe; 1 km W Andampibe, Cap Masoala; Parc National Mananara-Nord, 7.1 km 261° Antanambe; Res. Ambodiriana, 4.8 km 306°Manompana, along Manompana river; Ile Sainte Marie, Forêt Kalalao, 9.9 km 34° Ambodifotatra; Parcelle E3 Tampolo; Mahavelona (Foulpointe); Mahavelona (Foulpointe), Forest Andalava; Reserve Betampona, Camp Vohitsivalana, 37.1 km 338° Toamasina; Reserve Betampona, Camp Rendrirendry 34.1 km 332° Toamasina; F.C. Andriantantely; 6 km ESE Andasibe ( = Perinet); Province **Fianarantsoa**: Nat. Pk.Ranomafana, Miaranony Forest; Ranomafana Nat. Park, Valoloaka forest; Forêt d'Ambalagoavy Nord, Ikongo, Ambatombe; 45 km S. Ambalavao; Rés. Andringitra, 43 km S Ambalavao.

***Odontomachus simillimus*** Smith:

Figures: **worker** 12a,b, 13b; **queen** 12c,d; **male** 12e,f;

**Type material:**

*Odontomachus simillimus* Smith, 1858: 80 [31]. Type locality: Fiji Islands [not examined]. Junior synonym of *haematodus* by Roger, 1861: 24 [26]; revived from synonymy by Wilson, 1959: 499 [32].

*Odontomachus haematoda* var. *breviceps*, Crawley, 1915: 239 [33]. Type locality: Christmas Island, Australia (BMNH) [not examined]. Synonymized with *simillimus* by Brown, 1976: 106 [1].

*Odontomachus haematodes* var. *fuscipennis* Forel 1913: 19 [34].Type locality: Peradeniya, Sri Lanka (MNHB?) [not examined]. Synonymized with *simillimus* by Wilson, 1959: 499 [32].

*Ponera pallidicornis* Smith, F. 1860: 73 [35]. Type locality: Makassar, Celebes (BMNH) [not examined]. Synonymized with *simillimus* by Brown, 1976: 106 [1].

**Worker measurements:** maximum and minimum based on n = 10 from Madagascar: HL 2.33–2.63, HW (across vertex) 1.64–2.03, HW (across upper eye margin) 1.77–2.06, CI 75–81, EL 0.20–0.23, ML 1.14–1.28, MI 48–51, SL 2.16–2.43, SI 109–123, WL 2.62–3.06. FL 2.29–2.56, PW 1.02–1.24.

**Queen measurements:** maximum and minimum based on n = 5 from Madagascar: HL 2.37–2.55, HW (across vertex) 1.79–2.03, HW (across upper eye margin) 1.87–2.13, CI 79–84, EL 0.49–0.53, ML 1.17–1.30, MI 49–52, SL 2.15–2.38, SI 111–118, WL 3.13–3.19. FL 2.36–2.58.

**Male measurements:** maximum and minimum based on n = 1 from Madagascar: HL 0.89, HW 1.19, CI 133, EL 0.59, SL 0.19, SI 16, WL 2.44. FL 1.73.

**Worker diagnosis:** Workers and males are very similar in morphology and size to *troglodytes* Bivariate plots of metric measurements did not distinguish the two species. Workers and queen have fine, glossy dorsal striation on head and mesosoma. Metasternal process low and rounded (Fig. 13b). Metasternal process can be viewed in mounted specimens by removing a hind leg and coxa. Brown [1] provides a description and additional references.

**Figure 11:**
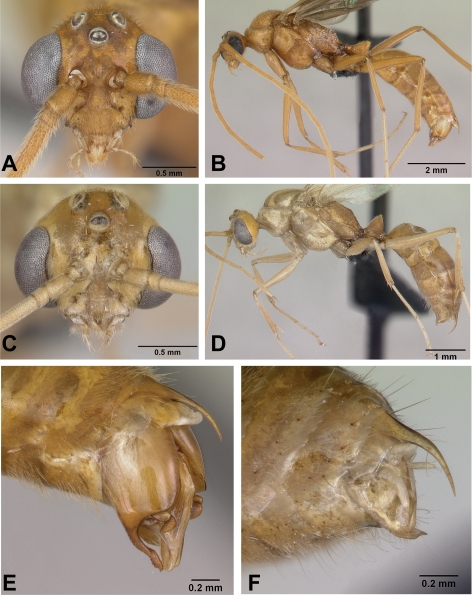
*Odontomachus* spp. males full face, lateral view, and oblique lateral view of terminalia. A, B, and E, *coquereli* CASENT0063858. C, D, and F, *troglodytes* CASENT0096412.

**Figure 12:**
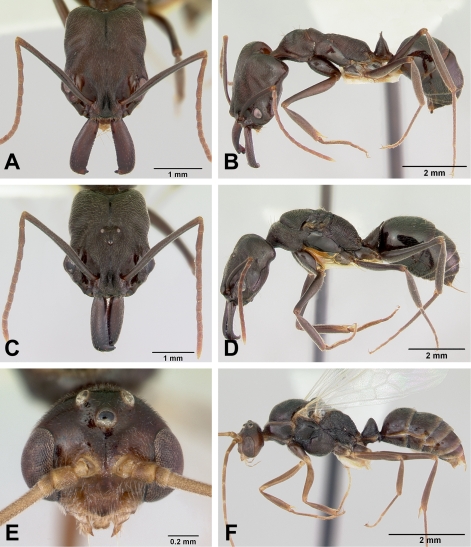
*Odontomachus simillimus* full face and lateral view. A–B, worker CASENT0172667. C–D, queen CASENT0172668. E–F, male CASENT0172666.

**Figure 13:**
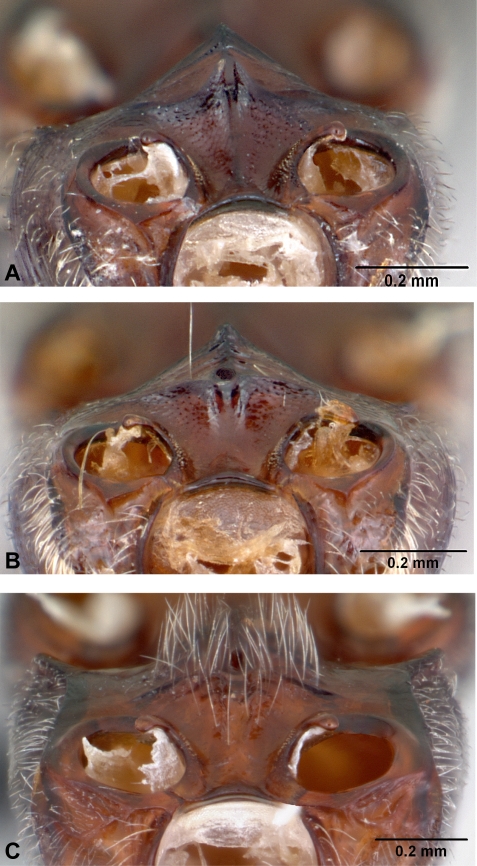
*Odontomachus* spp. ventral aspect of posterior mesosoma viewed from underneath and from rear with coxa and petiole removed to show metasternal process. A, *troglodytes* CASENT0009961. B, *simillimus* CASENT0009988. C, *coquereli* CASNET0009962.

**Figure 14:**
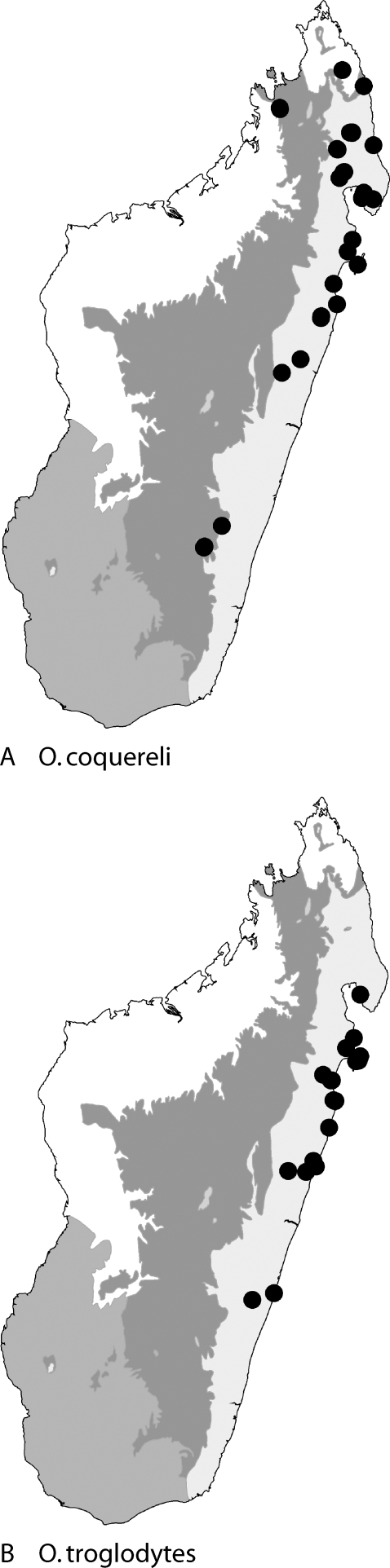
collection localities of *Odontomachus* in Madagascar. Map shows major ecoregions: east (light gray): rainforest, central (dark gray): montane forest; west (white): tropical dry forest; southwest (medium gray): desert spiny bush thicket.

**Distribution and biology.** Known though most of the literature as “*O. haematodes”* (Linnaeus) 1758 which is a different species. Forel's [25:159] record of “*O. haematodes*” from Seychelles, Mahé most likely refers to *simillimus*.

Found in clearings and secondary growth throughout the Indo-Pacific. The records from the Seychelles clearly represent an introduction. *O. simillimus* is not known from Madagascar and may have difficulty in establishing on Madagascar because of the presence of the morphologically and ecologically similar *O. troglodytes*.

**CO1.** The average within species CO1 divergence for *O. simillimus* was 3.212% with much variation between islands (Max 5.786, SE = 0.273). Importantly, although bivariate plots of worker measurements do not reliably separate *O. simillimus* from the ecologically similar *O. troglodytes*, the two species are, on average, 7–8% divergent within the CO1 barcode.

**Diagnostic barcoding loci.** *O. simillimus*: C-265, T-267, T-528.

**Specimens examined for *Odontomachus simillimus***: Additional details are provided for the specimens from Seychelles.

INDONESIA: Irian Jaya, Maffin Bay; PT. Freeport Concession, Siewa Camp; PAPUA NEW GUINEA: Los Negros, Admiralty Islands; Milne Bay, Morobe, Finschhafen, Biak Island; PHILIPPINES: Leyte, Tacloban; SEYCHELLES: Silhouette Island, Grande Barbe, 7/22–23/2000, J.Gerlach; Silhouette Island, Jardin Marron, 7/5/2000, J.Gerlach; SOLOMON ISLANDS: Kungana Bay, Rennell Island; Guadalcanal, Tenaru River; Kungana Bay, Rennell Island, Anuda Island; NW end of Bellona Island; Tevia Bay, Vanikoro Island, Santa Cruz Islands; Mohawk Bay, Matema Island, Santa Cruz Islands, Pavuvu, Russell Island; VANUATU: Espiritu Santo Island.

***Odontomachus troglodytes*** **Santschi**

Figures: **worker** 10e,f, 13a; **queen** 10g,h; **male** 11c,d,f; **map** 14b

**Type material:** *Odontomachus haematodes troglodytes* Santschi, 1914: 58 [36]. Lectotype worker: Kenya, Shimoni cave (NHMB), designated by Brown, 1976: 106 [2] [examined] AntWeb CASENT0101134. Raised to species Brown, 1976: 106 [1].

*Odontomachus haematodus stanleyi* Wheeler, 1922: 102 [37]. Type worker: DRC (Zaire) Stanleyville, 25° 10′E, 0°30′N Feb 1915, (AMNH) [examined] AntWeb CASENT0104653, CASENT0104654. Synonymized with *troglodytes* by Brown, 1976: 106 [1].

**Worker measurements:** maximum and minimum based on n = 15 from Madagascar: HL 2.23–2.66, HW (across vertex) 1.56–1.92, HW (across upper eye margin) 1.69–1.98, CI 74–78, EL 0.40–0.47, ML 1.13–1.33, MI 45–54, SL 2.07–2.42, SI 117–127, WL 2.61–3.07. FL 2.28–2.65, PW 1.02–1.19.

The specimens from Madagascar are notably smaller than specimens in CAS collection from South Africa, central Africa and Sao Tome. Maximum and minimum measurements based on n = 5: HL 2.52–2.94, HW (across vertex) 1.81–2.25, HW (across upper eye margin) 1.94–2.31, CI 74–79, EL 0.41–0.51, ML 1.19–1.38, MI 47–49, SL 2.24–2.53, SI 110–122, WL 2.88–3.23. FL 2.42–2.91, PW 1.13–1.36.

**Queen measurements:** maximum and minimum based on n = 5 from Madagascar: HL 2.59–2.74, HW (across vertex) 1.99–2.19, HW (across upper eye margin) 2.05–2.18, CI 78–79, EL 0.56–0.59, ML 1.39–1.44, MI 52–55, SL 2.36–2.52, SI 112–119, WL 3.18–3.49. FL 2.67–2.76.

**Male measurements:** maximum and minimum based on n = 5 from Madagascar: HL 1.00–1.04, HW 1.30–1.35, CI 127–133, EL 0.68–0.70, SL 0.22–0.26, SI 17–19, WL 2.52–2.59. FL 1.80–1.88

**Worker Diagnosis:** Workers of this species can be easily distinguished from *coquereli* by their smaller size, distinct extraocular furrows and temporal ridges on vertex and short and blunt mandibular teeth. Brown (1976) provides additional description and references.

**Distribution and biology.** *O. troglodytes* was first reported from Madagascar by André [38:290] as *O. haematodes* (Linnaeus). African and Malagasy records of *haematodes* actually refer to *troglodytes.* In Madagascar, *troglodytes* is widespread throughout the east in secondary habitats, including coastal scrub, eucalyptus plantations, littoral forest, and rainforest below 800 m elevation. This species is also widespread across sub-Saharan Africa in second growth forests and open habitats. Forel [25:159] recorded *Odontomachus* (as *haematodes*) from Seychelles. These specimens have not been examined but probably refer to *O*. *simillimus* and not *troglodytes*.

Because of its preference of secondary habitats, it is possible that *troglodytes* in Madagascar is a recent colonist from Africa, possibly introduced by humans. This is in contrast to *coquereli* which is most closely related to Melanesian species in the *tyrannicus* group.

Our collections in Madagascar were focused primarily on less disturbed habitats, thus the distribution map (Fig. 10b) probably does not reflect the full extent of its range. *O. troglodytes* was most often recorded nesting in rotten logs (30 collection records) followed by sifted litter (15). Males were collected at light, malaise traps, and yellow pan traps.

A lab colony was kept for a number of months and thrived on a diet of crickets, producing numerous larvae, brood, and males. The trap jaw behavior is very similar to that of *O. bauri* [39, Fisher unpublished]. When disturbed, the specimen use trap jaw propulsion to “jump” away.

**Figure 15:**
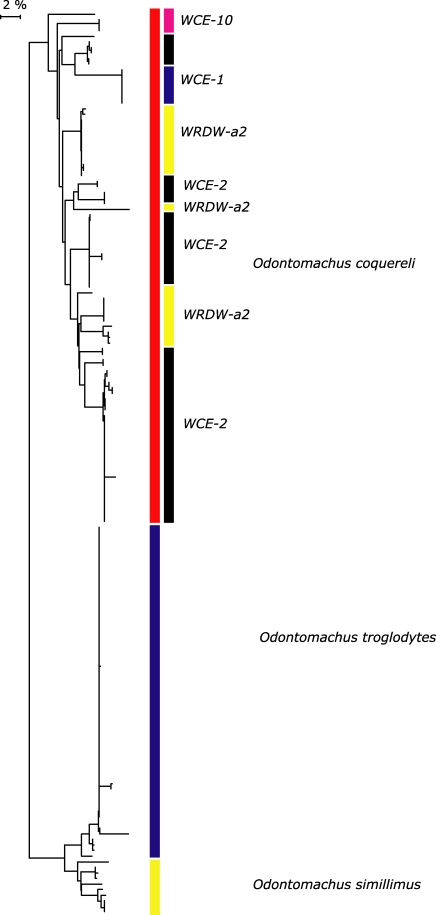
NJ tree of K2P for three species of *Odontomachus* in Madagascar and Africa (all specimens with >500 bp). Deep divergences evident between *coquereli, troglodytes,* and *simillimus* are evident. Deep divergences within *O. coquereli* are apparent. The rightmost column of colors differentiate which biogeographical groupings of Wilmé et al [29] these populations fall. WCE-1 = Binara. WCE-10 = Manongarivo. WCE-2 = Mahavelona, Kalalao, Betampona, Mananara-Nord, Marojejy, Anjanaharibe. WRDW-a2 = Akirindro, Ambanitaza, Anjanaharibe.

**CO1.** Shallow intraspecific and deep interspecific divergences between *O. troglodytes* in Madagascar and Africa and the other species – what one might expect if it has been recently introduced. Average within species sequence divergence of 0.4% (Figs 15, 17).

**Figure 16:**
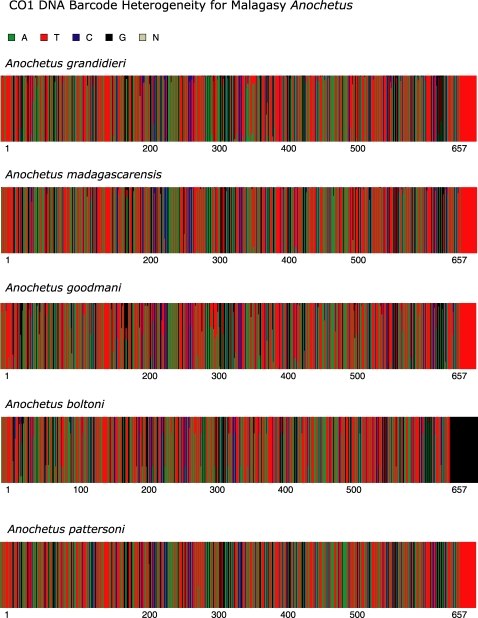
*Anochetus* spp. CO1 DNA barcode heterogeneity. *A. grandidieri* (n = 113), *A. madagascarensis* (n = 115), *A. goodmani* (n = 47), *A. boltoni* (n = 12) and *A. pattersoni* (n = 3).

**Figure 17:**
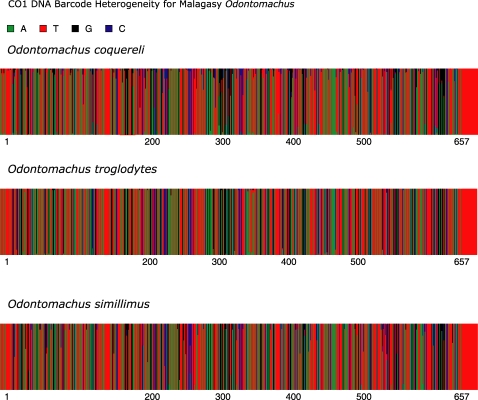
*Odontomachus* spp. CO1 DNA barcode heterogeneity. *O. coquereli* (n = 97), *O. troglodytes* (n = 53) and *O. simillimus* (n = 13).

**Diagnostic barcoding loci.** *O. troglodytes*: G-1659, G-465, G-519, T-535, A-537.

**Specimens examined for** ***Odontomachus troglodytes*** **:** Specimens from 105 separate collection events from the following 40 localities were examined. **CAMEROON**: Sud: Res. de Faune de Campo, 2.16 km 106° ESE Ébodjé; Sud-Ouest: Bimbia Forest, 7.4 km 119° ESE Limbe. **CENTRAL AFRICAN REPUBLIC**: Prefecture Sangha-Mbaéré: Parc National Dzanga-Ndoki, 39.6 km 174° S Lidjombo; Parc National Dzanga-Ndoki, 38.6 km 173° S Lidjombo; Parc National Dzanga-Ndoki, 37.9 km 169° S Lidjombo; Réserve Spéciale de Forêt Dense de Dzanga-Sangha, 12.7 km 326° NW Bayanga; Parc National Dzanga-Ndoki, Mabéa Bai, 21.4 km 53° NE Bayanga. **GABON**: Estuaire: Pointe Ngombe, Ekwata, 16 km 240° WSW Libreville; Libreville; F.C. Mondah, 21 km 331° NNW Libreville. **GABON**: Ogooue-Maritime: Aire d'Exploit. Rationnelle de Faune des Monts Doudou, 25.2 km 304° NW Doussala; Reserve de la Moukalaba-Dougoua, 12.2 km 305 NW Doussala; Reserve de Faune de la Moukalaba-Dougoua, 12.2 km 305° NW Doussala; Reserve de Faune de la Moukalaba-Dougoua, 10.8 km 214° SW Doussala; Woleu-Ntem: 31.3 km 108° ESE Minvoul; KENYA: [Côte d' Afrique or. angl. Shimoni; LIBERIA: Sapo Nat. Park. **MADAGASCAR**: **Toamasina**: Mahavelona ( = Foulpointe); 5.3 km SSE Ambanizana, Andranobe; Forêt d'Analava Mandrisy, 5.9 km 195° Antanambe; Res. Ambodiriana, 4.8 km 306°Manompana, along Manompana river; Ile Sainte Marie, Forêt Ambohidena, 22.8 km 44° Ambodifotatra; Ile Sainte Marie, Forêt Ampanihy, 14.4 km 52° Ambodifotatra; Ile Sainte Marie, Forêt Kalalao, 9.9 km 34° Ambodifotatra; Parcell K9 Tampolo; Tampolo; S.F. Tampolo, 10 km NNE Fenoarivo Atn.; Parcelle E3 Tampolo; Parcelle K7 Tampolo; Bridge at Onibi, NW of Mahavelona; Mahavelona (Foulpointe); 2.1 km 315° Mahavelona; Toamasina (Tamatave); Prison de Tamatave; Station forest de Tampolo, 10 km N Fenerive; Res. Betampona, Ambodiriana 45 km NW Toamasina; 10k N Brickaville; 11 km SE Ampasimanolotra ( = Brickaville); **Fianarantsoa**: Riv: Ranomafana Aff. de laroka; Local: Ranomafana RN2; Riv: laroka Aff de Rianila; Local: Manakana; Riv: Mahatsara Aff de Rianila; Local: Piste vers Brickaville; Riv: Rongaronga; Local: Ambodifaho; Riv: Rianila (Ivohitra); Local: Antseranambe; Riv: Santaravina; Local: Ampasipotsy-pont routier; Riv: Sandragniro; Local: Tanambao-Pont routier; Riv: Farimbogna; Local: Village 202 (Pont routier RN2); Riv: Ilazana; Local: Gri-gri; 8k E Kianjavato Vatovavy Forest; Ranomafana Nat. Park; 10k E Ranomafana; Ranomafana Nat. Park, 10 km E; Mananjary 2 km south; 7.6 km 122° Kianjavato, Forêt Classée Vatovavy; **SOUTH AFRICA**: **Mpumalanga**: Songimuelo Nat. Reserve, Kromdraai Camp, Komati River; **Natal**: Mtunzini; **Limpopo**: Dunstable Farm, 27 km E of Hoedspruit. **DEMOCRATIC REPUBLIC OF THE CONGO**: Stanleyville; Epulu.

### Complementary analyses to CO1

In some instances we chose to amplify independent nuclear markers to help interpret CO1 divergences involving populations where specimens were morphologically cryptic. Because of their high copy number and relatively conserved primer regions, we selected three ribosomal regions to amplify: 18S, 28S and ITS1. We had high expectations for the utility of these markers to complement the mtDNA barcode analysis based on our own experiences with other taxa [40], [41], the utility of these markers in other taxonomic groups where, for instance, ITS1 functions as a barcode [42], and, for 28S, based on predictions of others for the utility of this region as an alternative barcode region [43]. Unfortunately, we found that, while the CO1 data from species with exclusively (putatively) ergatoid queens had large phylogeographic signal, when compared to the three rRNA regions we utilized it was markedly simpler to generate, interpret and analyze. The rRNA markers utilized here, particularly 18S and 28S, can be useful for identifying interspecific (species as revised here) hybridization [see 40], [41], [43].

## Discussion

### The role of CO1 barcoding in taxonomic revision

In traditional morphology-based taxonomy, morphologically discrete forms are tentatively recognized and hypothesized to be species. Taxonomists search for consistent phenotypic discontinuities that may indicate the occurrence of reproductive isolation. Many ant species, however, show considerable geographical variation in morphological characters. An additional complication for morphology-based taxonomy is the difference between castes within the same species, e.g. males, major and minor workers, and queens. Sequence data provide an alternative set of characters to assist in inferring species boundaries. In addition, like morphological data, hypotheses can be evaluated in light of additional data on specimen distribution, biology, and behavior. In the example of *Anochetus* of Madagascar, sequence data impacted the taxonomic process at the following steps:

#### Caste association

Caste association, including male/female association, is a powerful contribution to taxonomic studies, especially for ants, which vary tremendously in morphology between sexes and castes. In this study, CO1 divergence was the principal source of data for revealing that small and large workers and queens are the same species. Though no morphological distinction in addition to size between the forms was noted, it remained unclear whether they belonged to the same species since no colony collection contained both size classes, even though they are often collected at the same site. One explanation is that small workers are produced by small queens. Small queens may represent an alternative reproductive strategy and may be only rarely produced by large queens. Further research will explore the reproductive biology of this species. The sequence data also confirmed the association of males collected in Malaise traps with the worker caste.

#### Type designation

The identities of many valid names are in question in Madagascar because insufficient geographic and morphological information was provided in their original descriptions, or type specimens are of uninformative minor worker castes or are damaged. For *Anochetus*, description of new species included the DNA barcode of a specimen from the paratype colony series to provide an additional tool for associating the name with type specimens. This facilitates linking the name to the type specimen if the identity of the type is called into question.

#### Evolutionary questions and biogeographic patterns

Sequencing revealed patterns of geographic coherence and divergence that were not revealed in morphological analysis. A good example of this is the deep divergence in isolated populations of *A. goodmani,* and *O. coquereli* (see *Species as hypotheses* below*)*. These results will direct future morphological and evolutionary studies on these divergent populations.

#### Identification

In-depth morphological study, a more time-consuming process than the DNA analysis undertaken in this study, was applied to outliers identified by the DNA analysis. For example, in the inventory described in part I, 22 collections of *Anochetus* from three species were included. All were correctly identified using sequence data. Specimens within the same species that showed high sequence divergence, however, were culled for morphological scrutiny (e.g. *A. madagascarensis* from Amato and Binara).

#### Biogeography

This combination of traditional taxonomy and DNA barcoding has produced a wealth of biogeographic hypotheses to be tested. Do more basal lineages have more restricted or wider distributions, compared to younger taxa? Are evident patterns of genetic isolation by distance within the ergatoid ponerines examined here shared by all those with wingless queens? Are the mechanisms of isolation the same? Do the phylogeographic groupings correspond with the Wilme *et al.* [29] biogeographic regions hypothesized largely as related to primates? Taxonomy has always had this style of iterative hypothesis testing, but adding an explicit molecular component as with DNA barcoding – allows these hypotheses to be more transparent.

### Species as hypotheses

The existence of any species is a hypothesis to be tested, and the transparency of species delimitation is one of the major additions that DNA barcoding brings to systematics. In our analysis, the deep sequence divergences within *A. goodmani* suggests that populations from the north and south of western Madagascar have a long history of isolation, and could in fact be separate species (Fig. 6). However, there are alternate hypotheses. This species has wingless queens. Species of ants that lack winged queens, reproduce by fission and have reduced dispersal ability, particularly when measured using a maternally inherited genetic marker. Thus, we might expect that those populations now restricted to isolated relict pockets of moist habitat in the dry west would show deep divergence [for example – 44–46], and represent distinct, evolutionarily significant units [47], if not distinct species. By contrast in *A. madagascarensis* and *A. grandidieri*, where only winged queens have been observed, within-species sequence divergences are much lower. We are currently testing the hypothesis that female-limited dispersal has caused the extremely site-specific phylogeographic signal by assaying nuclear genes. It is possible that these populations, separated at such a large spatial scale, will show strong genetic differentiation for both nuclear and mtDNA markers between localities [44]. The CO1 analysis does not unequivocally indicate that *A. goodmani* is more than one species, but it does suggest future hypotheses of species membership to be tested.

Molecular approaches to species identification have been criticized for potentially overestimating [48], [49], and/or underestimating biodiversity. Species diversity will be underestimated when collections include quickly evolving species-pairs [9] where interspecific divergences are less than or equal to intraspecific variation. Our data set contains one potential example of this phenomenon: individuals of *Anochetus goodmani* collected from Binara on the north east coast and Parc National de Kirindy Mite on the south west coast. Individuals from these populations are separated by, on average, 6.0% sequence divergence. Are these populations operating as separate species? Are these populations members of the same species but highly divergent? Our data alone cannot answer this question. But, of critical import, our data have identified a surprising level of within-species divergence and lays bare these differences to further study. A standard arthropod molecular clock for CO1 is 1.2–1.5% per million years [50]–[52]. In hymenopterans the rate for this gene is accelerated [53], and therefore average estimates should be interpreted with caution. However, the higher rates suggest that populations have been isolated for several hundred thousand years. The opportunity now exists to employ a suite of approaches (behavioral observations, tests of interbreeding, and phylogeographic resolution of more quickly evolving genetic markers) to test species membership.

### CO1 and complementary genetic analyses

Of all the molecular data used here, the CO1 data was by far the easiest to generate and interpret. While an inter-gene/genomic comparison of utility was not the intent of this research, we feel it important to comment on these differences here, while presenting a full multigene phylogeographic analysis of the covariance of genetic diversity and geographic separation in another manuscript. Within the context of a species description or revision, the relevant information here is that:

1. The CO1 barcode was very easy to generate. While the majority of specimens analyzed here are between 1–2 years old, we did generate full length barcodes (>600 bp) for specimens up to 14 years old. Barcodes were generated with the same primers and reaction conditions. Alternatively, rRNA data, variable at a species level, was often challenging to generate (i.e. sequence) due to long regions of t-repeats and uncharacterized intra-individual variation (in ITS1).
2. We found no evidence for Numts [54] or other misplaced nuclear markers that would introduce conflict into our analysis if not spotted.
3. CO1 sequences never showed intra-individual variation as did some of the rRNA markers.
4. Although the species described here (especially *O. coquereli, A. goodmani* and *A. boltoni*) contain large CO1 divergences, such variation is always geographically segregated, as one might expect from a species where the queens (when known) are ergatoid.

In the worst-case scenario, by describing species containing large intra-specific CO1 divergences, we have missed morphologically cryptic diversity within these species. However, the DNA data, collection records, measurements and photo-digital accessions are all preserved in publicly accessible databases, facilitating the testing (and potential refutation) of our one-species hypothesis in the traditional, iterative, process of alpha-taxonomy.

### Collaborative Taxonomy

Species inventories are essential for documenting global diversity and generating necessary material for taxonomic study. However, for inventories to be relevant in the short term, the taxonomic process must reduce the bottlenecks in describing and identifying specimens. The shear diversity of arthropods can easily overwhelm an inventory system with too many specimens, the bulk of which are outside the focal expertise of the taxonomists. As an example, the NSF-funded Arthropod Inventory of Madagascar has shipped over a third of million specimens to over 150 participating taxonomic collaborators [5]. Major taxonomic products from these inventories, which will take decades to produce, represent only a fraction of the diversity collected, and provide no short-term return of biodiversity data to Madagascar.

The development of “collaborative taxonomy” would permit researchers to participate collectively in an accelerated team-driven taxonomic process. Key participants in collaborative taxonomy are (i) inventory teams led by conservationists, ecologists, and taxonomists, (ii) traditional morphology-based taxonomists equipped with imaging tools, and (iii) geneticists. Under this plan, inventory teams would generate specimens and sequence data in collaboration with geneticists. Geneticists, in turn, would work directly with the taxonomist who identifies the need for additional sequencing of specimens. Taxonomists would then combine extensive sequencing data with their morphological and ecological analysis, assisted by new technologies in digital imaging and web-based delivery (e.g. www.antweb.org and www.barcodinglife.org), to infer species limits and frame evolutionary context for species.

Nothing can replace the countless hours of careful observation necessary to understand variation and to delimit species boundaries. However, the addition of sequence data provides a means to create short-term results from inventories and at the same time generate data helpful to taxonomists. For taxonomists, sequencing highlights the specimens most deserving of focused study. We tested this collaborative model by revising the ant genera *Anochetus* and *Odontomachus* of Madagascar using a combination of morphological and genetic character sets based on inventories in Madagascar.

### Future

This study demonstrates how sequence data, combined with morphological analysis and innovations in imaging and web delivery, have set the stage for accelerated discovery and documentation of global species diversity. The combination of DNA sequence data with inventory and traditional taxonomy is a model that can be applied across disciplines and will allow analytical needs to scale to the enormity of the biodiversity crisis [55]. It will help in the identification and conservation of the evolutionary processes that generate and preserve biodiversity.

Little time remains to document and protect global biodiversity. Taxonomists, equipped with modern tools and collaborations, have a chance to move systematics to the forefront of conservation and the public's attention. With increased taxonomic output and improved public access and visibility, public support for the discovery of life on this planet will follow.

## Supporting Information

Appendix S1 Accessions and collection information for all sequences created in this study.
(0.64 MB XLS)

Appendix S2 This is the current article provided as a TaxonX XML document. TaxonX models taxonomic treatments and allows semantic enhancement so machines can understand, mine and extract the content (see http://plazi.org).
(0.11 MB RTF)
